# Prognostic Implications of Lateral Lymph Nodes in Rectal Cancer: A Population-Based Cross-sectional Study With Standardized Radiological Evaluation After Dedicated Training

**DOI:** 10.1097/DCR.0000000000002752

**Published:** 2023-06-01

**Authors:** Tania C. Sluckin, Eline G.M. van Geffen, Sanne-Marije J.A. Hazen, Karin Horsthuis, Regina G.H. Beets-Tan, Corrie A.M. Marijnen, Pieter J. Tanis, Miranda Kusters

**Affiliations:** 1 Department of Surgery, Amsterdam UMC location Vrije Universiteit Amsterdam, Amsterdam, The Netherlands; 2 Cancer Center Amsterdam, Treatment and Quality of Life, Amsterdam, The Netherlands; 3 Cancer Center Amsterdam, Imaging and Biomarkers, Amsterdam, The Netherlands; 4 Department of Radiology, Amsterdam UMC location Vrije Universiteit Amsterdam, Amsterdam, The Netherlands; 5 Department of Radiology, The Netherlands Cancer Institute, Amsterdam, The Netherlands; 6 GROW School for Oncology and Developmental Biology, University of Maastricht, Maastricht, The Netherlands; 7 Department of Radiology, University of Southern Denmark, Odense University Hospital, Odense, Denmark; 8 Department of Clinical Research, University of Southern Denmark, Odense University Hospital, Odense, Denmark; 9 Department of Radiation Oncology, Leiden University Medical Center, Leiden, The Netherlands; 10 Department of Radiation Oncology, The Netherlands Cancer Institute, Amsterdam, The Netherlands; 11 Department of Surgery, Amsterdam UMC location University of Amsterdam, Amsterdam, The Netherlands; 12 Department of Surgical Oncology and Gastrointestinal Surgery, Erasmus Medical Center, Rotterdam, The Netherlands

**Keywords:** Lateral lymph nodes, MRI re-review, Rectal cancer

## Abstract

**BACKGROUND:**

There is an ongoing discussion regarding the prognostic implications of the presence, short-axis diameter, and location of lateral lymph nodes.

**OBJECTIVE:**

To analyze lateral lymph node characteristics, the role of downsizing on restaging MRI, and associated local recurrence rates for patients with cT3–4 rectal cancer after MRI re-review and training.

**DESIGN:**

Retrospective population-based cross-sectional study.

**SETTINGS:**

This collaborative project was led by local investigators from surgery and radiology departments in 60 Dutch hospitals.

**PATIENTS:**

A total of 3057 patients underwent rectal cancer surgery in 2016: 1109 had a cT3–4 tumor located ≤8 cm from the anorectal junction, of whom 891 received neoadjuvant therapy.

**MAIN OUTCOME MEASURES:**

Local recurrence and (ipsi) lateral local recurrence rates.

**RESULTS:**

Re-review identified 314 patients (35%) with visible lateral lymph nodes. Of these, 30 patients had either only long-stretched obturator (n = 13) or external iliac (n = 17) nodes, and both did not lead to any lateral local recurrences. The presence of internal iliac/obturator lateral lymph nodes (n = 284) resulted in 4-year local recurrence and lateral local recurrence rates of 16.4% and 8.8%, respectively. Enlarged (≥7 mm) lateral lymph nodes (n = 122) resulted in higher 4-year local recurrence (20.8%, 13.1%, 0%; *p* <.001) and lateral local recurrence (14.7%, 4.4%, 0%; *p* < 0.001) rates compared to smaller and no lateral lymph nodes, respectively. Visible lateral lymph nodes (HR 1.8 [1.1–2.8]) and enlarged lateral lymph nodes (HR 1.9 [1.1–3.5]) were independently associated with local recurrence in multivariable analysis. Enlarged lateral lymph nodes with malignant features had higher 4-year lateral local recurrence rates of 17.0%. Downsizing had no impact on lateral local recurrence rates. Enlarged lateral lymph nodes were found to be associated with higher univariate 4-year distant metastasis rates (36.4% vs 24.4%; *p* = 0.021), but this was not significant in multivariable analyses (HR 1.3 [0.9–1.]) and did not worsen overall survival.

**LIMITATIONS:**

This study was limited by the retrospective design and total number of patients with lateral lymph nodes.

**CONCLUSIONS:**

The risk of lateral local recurrence due to (enlarged) lateral lymph nodes was confirmed, but without the prognostic impact of downsizing after neoadjuvant therapy. These results point toward the incorporation of primary lateral lymph node size into treatment planning. See Video Abstract.

**IMPLICACIONES PRONÓSTICAS DE LOS NÓDULOS LINFÁTICOS LATERALES EN EL CÁNCER DE RECTO: UN ESTUDIO TRANSVERSAL DE BASE POBLACIONAL CON EVALUACIÓN RADIOLÓGICA ESTANDARIZADA DESPUÉS DE UN ENTRENAMIENTO ESPECÍFICO:**

**ANTECEDENTES:**

Hay una discusión en curso acerca de las implicaciones pronósticas de la presencia, el diámetro del eje corto y la ubicación de los nódulos linfáticos laterales.

**OBJETIVO:**

Analizar las características de los nódulos linfáticos laterales, el rol de la reducción de tamaño en la IRM de reestratificación y las tasas de recurrencia local asociadas para pacientes con cáncer de recto cT3-4 después de una nueva revisión y entrenamiento de IRM.

**DISEÑO:**

Estudio transversal retrospectivo poblacional.

**CONFIGURACIÓN:**

Este proyecto colaborativo fue dirigido por investigadores locales de los departamentos de cirugía y radiología en 60 hospitales holandeses.

**PACIENTES:**

3057 pacientes fueron operados de cáncer de recto en 2016: 1109 tenían tumor cT3-4 ubicado a ≤8 cm de la unión anorrectal de los cuales 890 recibieron terapia neoadyuvante.

**INTERVENCIONES(S):**

Ninguna.

**PRINCIPALES MEDIDAS DE RESULTADO:**

recurrencia local y tasas de recurrencia local ipsilateral.

**RESULTADOS:**

Una nueva revisión identificó a 314 pacientes (35%) con nódulos linfáticos laterales visibles. 30 de estos pacientes tenían solo nódulos obturadores estirados (n = 13) o ilíacos externos (n = 17) y ambos no provocaron recurrencias locales laterales. La presencia de nódulos linfáticos laterales ilíacos internos/obturadores (n = 284) dio como resultado tasas de recurrencia local y recurrencia local lateral a los 4 años del 16.4% y el 8.8%, respectivamente. Los nódulos linfáticos laterales agrandados (≥7 mm) (n = 122) resultaron en una mayor recurrencia local a los 4 años (20.8%, 13.1%, 0%, *p* < 0.001) y recurrencia local lateral (14.7%, 4.4%, 0%, *p* < 0.001) en comparación con nódulos linfáticos más pequeños y sin nódulos linfáticos laterales, respectivamente. Los nódulos linfáticos laterales visibles (índice de riesgo 1,8 (1,1–2,8)) y los nódulos linfáticos laterales agrandados (índice de riesgo 1.9 (1.1–3.5)) se asociaron de forma independiente con la recurrencia local en el análisis multivariable. Los nódulos linfáticos laterales agrandados con características malignas tuvieron tasas de recurrencia local lateral a 4 años más altas del 17.0%. La reducción de tamaño no tuvo impacto en las tasas de recurrencia local lateral. Los nódulos linfáticos laterales agrandados se asociaron con tasas univariadas más altas de metástasis a distancia a los 4 años (36.4%, 24.4%, *p* = 0.021), pero no en el análisis multivariable (índice de riesgo 1.3 (0.9–1.8)), y no empeoró la supervivencia general.

**LIMITACIONES:**

Este estudio estuvo limitado por el diseño retrospectivo y el número total de pacientes con nódulos linfáticos laterales.

**CONCLUSIONES:**

Se confirmó el riesgo de recurrencia local lateral debido a los nódulos linfáticos laterales (agrandados), pero sin el impacto pronóstico de la reducción después de la terapia neoadyuvante. Estos resultados apuntan hacia la incorporación del tamaño del nódulo linfático lateral primario en la planificación del tratamiento. *(Traducción—Dr. Aurian Garcia Gonzalez*)

The adoption of adequate neoadjuvant therapy followed by total mesorectal excision surgery has helped reduce overall local recurrence (LR) rates for patients with locally advanced rectal cancer.^1–3^ However, despite an absolute reduction, there has been a proportional increase in lateral LRs (LLRs), most likely due to inadequate treatment of lateral lymph nodes (LLNs).^4^ LLNs are situated outside the mesorectum and are not removed during standard total mesorectal excision surgery.

An international guideline for the appropriate treatment of LLNs is lacking. The recent, large-scale Lateral Node Consortium Study investigated oncological outcomes for patients with LLNs and suggested that ≥7 mm (short axis [SA]) LLNs should be considered clinically suspicious. These enlarged LLNs resulted in a 5-year LLR rate of 19.5%.^5^ Furthermore, internal iliac LLNs remaining >4 mm and obturator LLNs remaining >6 mm on restaging MRI had 5-year LLR rates of 52.3% and 17.8%, respectively, whereas LLNs that shrunk below these thresholds resulted in 0% LLR.^6^ This suggests that primary and restaging sizes are needed to make appropriate treatment decisions and that internal iliac LLNs had the highest absolute risk.

Oncological outcomes for primarily, and persistently, enlarged LLNs found by the Consortium study require validation. In addition, the role of malignant features (heterogeneity, irregular border, loss of fatty center, and round shape) is still unclear; although Ogura et al^6^ found no significant association, Kroon et al^7^ indicated a role for malignant features in smaller LLNs.

The objective was to analyze the prognostic implications of LLNs in patients with cT3–4 rectal cancer ≤8 cm from the anorectal junction (ARJ) after standardized MRI re-review and dedicated training.

## MATERIALS AND METHODS

This population-based, cross-sectional cohort study examined all patients treated for primary rectal cancer between January 1 and December 31, 2016, in the Netherlands. A data set of these patients was registered in the Dutch ColoRectal Audit during that period and was expanded with additional variables collected between October 15, 2020, and February 28, 2022. A similar method is described elsewhere.^8^ Each participating hospital formed a team of collaborators from surgery, radiology, and radiation oncology departments.

**FIGURE 1.**
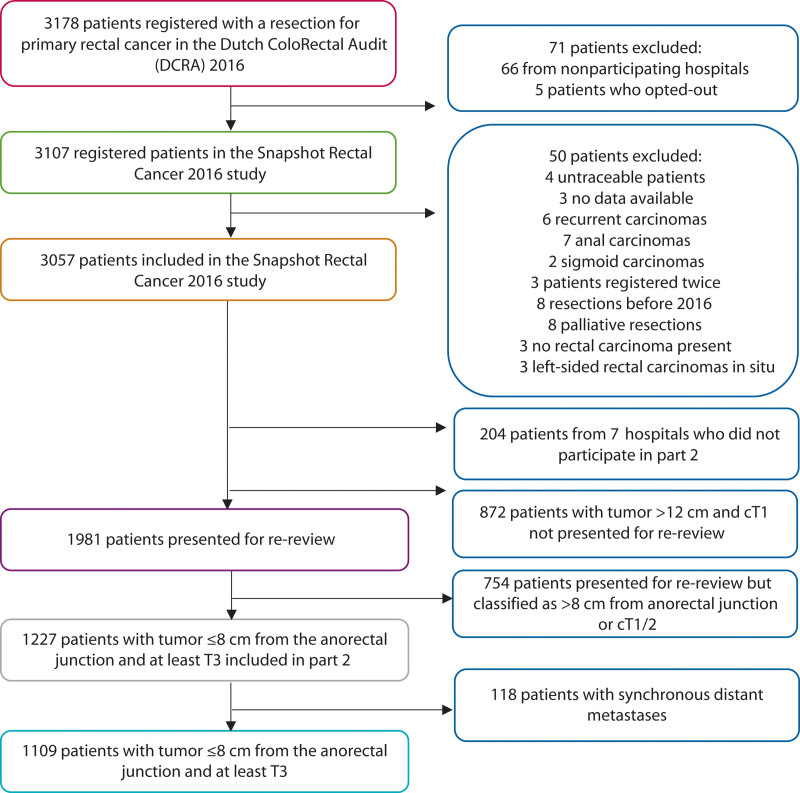
Flow chart of participants.

In part 1, the surgical team from each hospital recorded diagnostic, therapeutic, and follow-up variables based on patient medical record review. All data were verified centrally once the collection was completed. The medical records of a subset of patients with ≥cT2 stage rectal cancer ≤12 cm from the ARJ based on available MRIs were extracted for review by the local participating radiologist(s). Based on MRI re-review, patients with a tumor ≤8 cm from the ARJ and ≥cT3 stage were included. Due to their influence on oncological outcomes, patients with synchronous metastases (≤3 months) were excluded (Fig. 1). Appendix 1 at https://links.lww.com/DCR/C201 provides details regarding data management and privacy.

### Preassessment Training

One or 2 consultant abdominal radiologist(s) per hospital participated in part 2 of this study and underwent a dedicated 2-hour training session regarding LLNs, provided by expert radiologists (K.H., R.G.H.B.-T.) with 17 and 24 years of experience, respectively. Significant improvements were seen in measurements and anatomical classifications of LLNs after training.^9^ During this training, the color atlas by Ogura et al^5,6^ was explained in detail. Lateral compartments were defined as follows: the lateral border of the main trunk of the internal iliac artery separates the obturator compartment (lateral) from the internal iliac compartment (medial). Once the internal iliac artery exits the pelvis, all remaining lymphatic tissue is considered as obturator compartment. External iliac LLNs were located ventral of the external iliac vessels. Afterward, participants received an additional 23-minute webinar describing the definitions of LLNs and regarding MRI-detected extramural venous invasion (mrEMVI) and tumor deposits.

After this, the re-review commenced. A color atlas of an entire rectal MRI depicting the lateral compartments was created by the study team and distributed for use during re-review (see Appendix 2 at https://links.lww.com/DCR/C202). Participants reported LLN details such as the primary and restaging SA diameter, location according to the aforementioned definitions, and whether malignant features were present. If applicable, imaging of an (L)LR was also reviewed. The central coordinating researcher was often physically present to support the MRI re-review.

### Outcome Analysis

**FIGURE 2.**
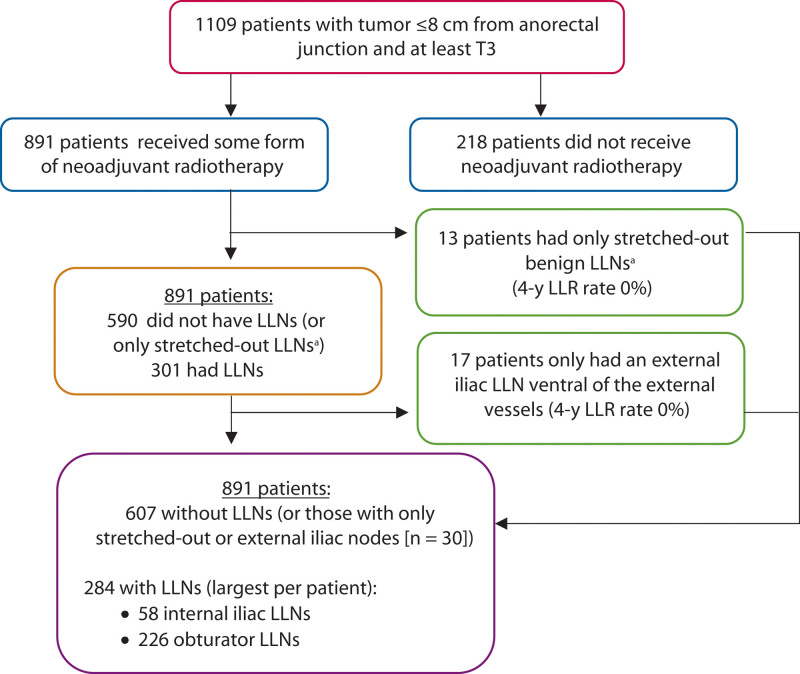
Continuation of the flowchart of participants. ^a^Stretched out benign LLNs are a subtype of obturator LLNs, dorsal of external iliac vessels in which the long axis is at least twice the length of the short axis with a maximum short axis diameter of 5 mm, with no malignant features present, and no change or increase in the restaging MRI. LLN = lateral lymph node.

Analyses were structured as follows (Fig. 2). Patients were divided into those who received neoadjuvant treatment (5 × 5 Gy or 25 × 2 Gy radiation dose with concomitant oral capecitabine [825 mg/m^2^]), which is generally considered to be an essential treatment for patients with LLNs, and those who did not. The main outcome parameters were 4-year LR and lateral LR (LLR) rates. Secondary outcomes were 4-year distant metastases (DM) and overall survival (OS). For all analyses, the largest LLN ipsilateral to the LLR was used. One patient developed an LLR on the contralateral side of the LLN because of a tumor deposit at the circumferential resection margin (CRM). This patient was classified as developing LR, but not LLR due to LLN. Analyses were first performed for all present LLNs and then for enlarged LLNs. Enlarged LLNs were defined as SA ≥7 mm.

Then, all patients with visible LLNs on MRI (internal iliac, external iliac, and obturator) were examined. Stretched-out obturator nodes were considered benign in a study by Ogura et al^5^ and were not included for analyses in the Consortium study. Therefore, these LLNs (defined as SA <5.0 mm, long axis at least twice the length of the SA, without malignant features or growth on restaging MRI) were also evaluated separately.

After this, clinically relevant LLNs (internal iliac/obturator LLNs) were analyzed further based on the anatomical location of their largest LLN. Patients with only external iliac or stretched-out obturator nodes were analyzed in the “no LLN” group. For these analyses, outcomes were examined per location (internal iliac/obturator) and according to size (<5.0, 5.0–6.9, ≥7.0). In addition, the influence of downsizing on restaging MRI according to cutoff values (≤4 mm internal and ≤6 mm obturator) and the presence and influence of malignant features on primary MRI were evaluated.^5,6^

### Statistics

Analyses were conducted in SPSS Statistics, version 26.0 (SPSS, Chicago, IL). Categorical data are presented as numbers with percentages and continuous variables as means with SDs or medians with an interquartile ranges. Subgroups were analyzed using the χ^2^ test, the Fisher exact probability test, or the independent *t* test. Univariable analysis identified predictors of (L)LRs and included visible LLN(s), enlarged (≥7 mm) LLN(s), location (internal iliac, external iliac, obturator), malignant features, and restaging diameters. Overall (L)LR, DM, and survival rates were analyzed with Kaplan-Meier analysis and compared with the log-rank test. The multivariable Cox regression model examined covariates with a *p* value of <0.10 from univariable analysis to determine independent associations of LLN characteristics with LR. This could not be performed for LLR due to low event rates. Surgical treatment of LLNs was not routine practice and was only incidentally performed without standardized technique; therefore, LLN surgery was not included in the prognostic models (details of patients who underwent LLN surgery are described elsewhere)^10^. Statistical significance was set at a *p* value of <0.05.

### Ethics

Central approval was obtained by the ethics board of Amsterdam UMC, the Netherlands, on June 30, 2020. Local approval from each participating center was obtained before the study commenced. Each center decided whether their patients provided written informed consent or were given the opportunity to opt out of the study.

## RESULTS

Sixty-seven of the 69 Dutch hospitals providing rectal cancer care in 2016 participated in this study, resulting in 3107 of 3178 eligible patients (97.8%; Fig. 1). Of the 3057 patients included in part 1, 60 hospitals participated in part 2 and resulted in 1109 patients (Table 1, Fig. 2). Median follow-up was 48 months (interquartile range, 26–54 months).

**TABLE 1.** Baseline characteristics of patients with cT3–4M0 rectal cancer located ≤8 cm from the anorectal junction based on MRI re-review (n = 1109) of those who received neoadjuvant radiotherapy (n = 891) and those who did not (n = 218)

*Variable*	*n = 891 (%*)	*n = 218 (%*)
Sex: male	581 (65.2)	157 (72.0)
Age, y, mean (SD)	72.1 (10.6)	74.9 (11.3)
Distance of tumor from anorectal junction, cm, mean (SD)	3.3 (2.5)	4.1 (2.3)
Tumor according to LOREC criteria On/below Above	541 (60.7) 350 (39.3)	104 (47.7) 114 (52.3)
Clinical T stage T3a (<1 mm beyond muscularis propria) T3b (1–4.9 mm beyond muscularis propria) T3c (5–15 mm beyond muscularis propria) T3d (>15 mm beyond muscularis propria) T4a (invasion of peritoneum) T4b (invasion surrounding organs/structures)	174 (19.5) 287 (32.2) 221 (24.8) 56 (6.3) 53 (6.0) 100 (11.2)	87 (39.9) 91 (41.7) 30 (13.8) 2 (0.9) 7 (3.2) 1 (0.5)
Threatened MRF or T4 on primary MRI (tumor ≤1 mm of the MRF)	439 (49.3)	32 (14.7)
Mesorectal clinical N stage N0 N1 N2	183 (20.5) 400 (44.9) 308 (34.6)	167 (76.6) 46 (21.1) 5 (2.3)
mrEMVI	314 (35.2)	32 (14.7)
Tumor deposits on primary MRI	143 (16.0)	4 (1.8)
All LLNs visible on primary MRI Largest LLN in obturator compartment Largest LLN in internal iliac compartment Patients with LLNs only in external iliac compartment Patients with only stretched-out “benign” obturator LLNs	314/891 (35.2) 226/314 (72.0) 58/314 (18.5) 17/314 (5.4) 13/314 (4.1)	58/218 (26.6) 40/58 (69.0) 8/58 (13.8) 10/58 (17.2) –
LLN characteristics One or more internal iliac/obturator LLN with SA ≥7 mm Any LLN with at least 1 malignant feature	122 (13.7) 157 (17.6)	6 (2.8) 18 (8.3)
Neoadjuvant treatment None Short-course radiotherapy Chemoradiotherapy Chemotherapy alone	– 338 (37.9) 553 (62.1) –	216 (99.1) ––2 (0.9)
Resection of primary tumor Local excision^a^ Local excision followed by TME Low anterior resection/TME Abdominoperineal resection Hartmann procedure Proctocolectomy Total exenteration	5 (0.6) – 411 (46.1) 338 (37.9) 134 (15.0) 2 (0.2) 1 (0.1)	7 (0.5) 2 (0.2) 123 (56.9) 49 (22.7) 34 (15.7) 1 (0.1) –
Underwent some form of additional surgery for LLN Yes No	33 (3.7) 858 (96.3)	– –
Resection margins (%) R0 R1	823 (92.4) 68 (7.6)	206 (94.5) 12 (5.5)

Data presented as n (%) or n/N (%) unless otherwise noted.

LLN = lateral lymph node; LOREC = low rectal cancer development program—lower border of the tumor is located beneath the attachment of the levator ani (seen on coronal plane); mrEMVI = MRI-identified extramural venous invasion; MRF = mesorectal fascia; PME = partial mesorectal excision; SA = short axis; TME = total mesorectal excision.

a Tumors were initially staged higher according to clinical staging but were later confirmed lower by pathology.

### Non-irradiated Patients

In total, 218 of 1109 patients (19.7%) with low, locally advanced rectal cancer did not receive any form of neoadjuvant radiotherapy. According to re-review, LLNs were present in 58 of these patients (26.6%). Eleven LLNs were ≥7 mm (19.0%): 6 internal iliac/obturator LLNs (54.5%) and 5 external LLNs (45.5%). Three patients (all with an enlarged internal iliac LLN) developed an LLR.

### Oncological Outcomes for External, Internal Iliac, and Obturator Nodes

**FIGURE 3.**
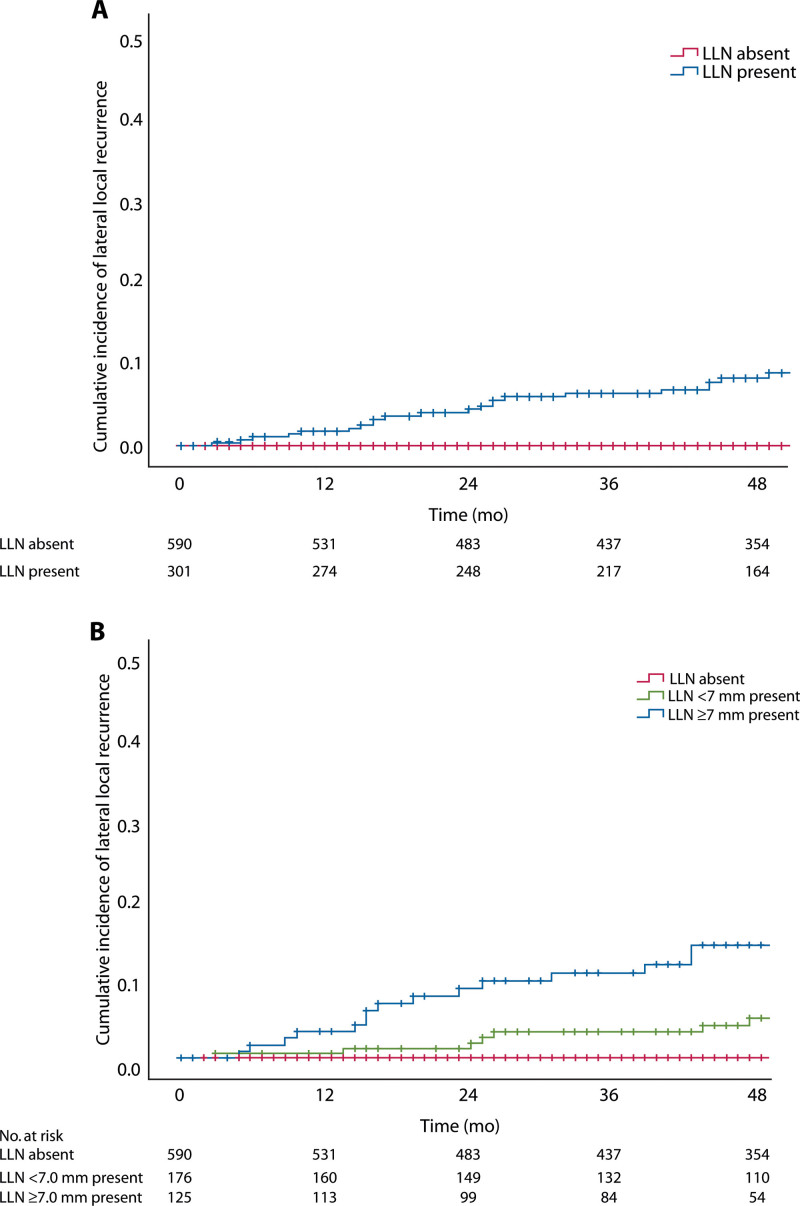
A, Lateral local recurrence rates for the presence of external, internal iliac, and obturator LLNs. B, Enlarged (≥7 mm) external, internal iliac, and obturator LLNs. LLN = lateral lymph node.

A total of 891 of 1109 patients (80.3%) received neoadjuvant radiotherapy, of whom 301 patients (33.8%) had visible internal iliac, external iliac, or obturator nodes. LLNs increased the LR rate from 7.2% to 15.8% (*p* < 0.001), LLR rate from 0.0% to 8.2% (*p* < 0.001; Fig. 3A), and DM rate from 24.6% to 32.5% (*p* = 0.029), compared to those without LLNs. Enlarged LLNs (n = 125) further increased the LR, LLR, and DM rates, respectively, to 20.2% (*p* < 0.001), 14.3% (*p* < 0.001; Fig. 3B), and 35.4% (*p* = 0.044).

### “Stretched-Out” and External Iliac Nodes

Thirteen patients only had “stretched-out” obturator LLNs. One patient developed an LR on the rectal stump (4-year LR 11.1%) and no LLRs occurred. Seventeen patients had only visible external iliac LLNs; 1 patient developed an anterior pelvic LR, encroaching on both vesicles and the bladder (4-year LR 6.2%), but no LLR (Fig. 2; see Appendix 3 at https://links.lww.com/DCR/C203).

### Oncological Outcomes for Internal Iliac and Obturator Nodes

Patients with internal iliac or obturator LLNs (n = 284, 31.9%; see Appendix 4 at https://links.lww.com/DCR/C204) had 4-year LR rates of 16.4% versus 7.0% for those without LLNs (*p* < 0.001) and 4-year LLR rates of 8.8% and 0%, respectively (*p* < 0.001). Present LLNs remained independently associated with an increased LR risk in multivariable analysis (HR 1.787; 95% CI, 1.130–2.827; *p* = 0.013; Table 2).

**TABLE 2.** Univariable and multivariable analyses of local recurrence in 891 patients with cT3–4 rectal cancer ≤8 cm from the anorectal junction who were treated with neoadjuvant radiotherapy (short course or chemoradiotherapy)

*Variable*	*No.*	*Univariable analysis*			*Multivariable analysis*		
		*HR*	*95% CI*	*p*	*HR*	*95% CI*	*p*
LLN of any size present No Yes	607 284	1 2.414	1.556–3.744	**<0.001**	1 1.787	1.130–2.827	**0.013**
Sex Male Female	581 310	1 0.953	0.600–1.515	0.839			
Age, y <55 55–75 ≥75	50 472 369	1 0.849 1.014	0.336–2.147 0.396–2.598	0.736			
Neoadjuvant radiotherapy 5 × 5 CRT	338 553	1 1.496	0.922–2.428	0.103			
Clinical T stage T3a T3b T3c T3d T4a T4b	174 287 221 56 53 100	1 1.474 2.138 2.637 5.913 4.460	0.645–3.367 0.941–4.854 0.915–7.601 2.378–14.704 1.907–10.430	**<0.001**	1 0.994 1.085 0.920 2.484 1.155	0.427–2.312 0.463–2.546 0.304–2.782 0.942–6.549 0.453–2.941	0.231
Mesorectal clinical N stage N0 N1 N2	183 400 308	1 0.669 1.618	0.349–1.281 0.897–2.917	**0.003**	1 0.641 1.208	0.327–1.255 0.646–2.258	0.059
mrEMVI Absent Present	577 314	1 2.543	1.635–3.956	**<0.001**	1 2.102	1.283–3.444	**0.003**
Tumor deposits Absent Present	748 143	1 2.569	1.592–4.146	**<0.001**	1 1.676	0.986–2.850	0.056
Surgery^a^ Sphincter nonsparing Sphincter sparing	340 551	1 0.508	0.328–0.789	**0.003**	1 0.580	0.358–0.940	**0.027**
Margin status R0 R1	823 68	1 6.837	4.176–11.193	**<0.001**	1 5.820	3.410–9.932	**<0.001**

These patients were included in the analysis based on visibility on primary staging MRI, independent of size.

Boldface indicates statistically significant findings.

LLN = lateral lymph node; mrEMVI = MRI-identified extramural venous invasion.

a Sphincter nonsparing includes abdominoperineal resections and proctocolectomy cases and sphincter sparing cases include (low) anterior resections and local excisions.

Explorative univariable analyses were performed to determine the most appropriate cutoff value for the SA diameter of LLNs (see Appendix 4 at https://links.lww.com/DCR/C204). Enlarged LLNs (≥7.0 mm, n = 122) were associated with significantly higher 4-year LR and LLR rates compared to <7.0 mm (n = 162) or no visible LLNs (n = 607), respectively (LR 20.8%, 13.1%, 7.0%: *p* < 0.001; LLR 14.7%, 4.4%, 0%: *p* < 0.001; Fig. 3). LLNs ≥7.0 mm remained a significant predictor of LR in multivariable analyses (HR 1.948; 95% CI, 1.085–3.495; *p* = 0.041; Table 3).

**Table 3.** Univariate and multivariate analyses of local recurrence in 891 patients with cT3–4 rectal cancer ≤8 cm from the anorectal junction who were treated with neoadjuvant radiotherapy (short course or chemoradiotherapy)

*Variable*	*No.*	*Univariable analysis*			*Multivariable analysis*		
		*HR*	*95% CI*	*p*	*HR*	*95% CI*	*p*
Enlarged (≥7 mm) LLN No LLN ≥7 mm <7 mm	607 122 162	1 3.017 1.979	1.784–5.101 1.152–3.401	**<0.001**	1 1.948 1.669	1.085–3.495 0.962–2.898	**0.041**
Sex Male Female	581 310	1 0.953	0.600–1.515	0.839			
Age, y <55 55–75 ≥75	50 472 369	1 0.849 1.014	0.336–2.147 0.396–2.598	0.736			
Neoadjuvant radiotherapy 5 × 5 CRT	338 553	1 1.496	0.922–2.428	0.103			
Clinical T stage T3a T3b T3c T3d T4a T4b	174 287 221 56 53 100	1 1.474 2.138 2.637 5.913 4.460	0.645–3.367 0.941–4.854 0.915–7.601 2.378–14.704 1.907–10.430	**<0.001**	1 0.987 1.080 0.912 2.474 1.105	0.424–2.297 0.460–2.535 0.301–2.760 0.938–6.525 0.424–2.878	0.227
Mesorectal clinical N stage N0 N1 N2	183 400 308	1 0.669 1.618	0.349–1.281 0.897–2.917	**0.003**	1 0.642 1.185	0.328–1.259 0.630–2.229	0.077
mrEMVI Absent Present	577 314	1 2.543	1.635–3.956	**<0.001**	1 2.115	1.290–2.467	**0.003**
Tumor deposits Absent Present	748 143	1 2.569	1.592–4.146	**<0.001**	1 1.715	0.999–2.942	0.050
Surgery^a^ Sphincter nonsparing Sphincter sparing	340 551	1 0.508	0.328–0.789	**0.003**	1 0.584	0.360–0.949	**0.030**
Margin status R0 R1	823 68	1 6.837	4.176–11.193	**<0.001**	1 5.767	3.370–9.870	**<0.001**

LLNs were included in the analysis based on visibility on primary staging MRI, and stratified for short-axis diameter with a cutoff value of 7 mm.

Boldface indicates statistically significant findings.

LLN = lateral lymph node; mrEMVI = MRI-identified extramural venous invasion.

a Sphincter nonsparing includes abdominoperineal resections and proctocolectomy cases and sphincter sparing cases include (low) anterior resections and local excisions.

Higher 4-year univariate DM rates were found for patients with enlarged LLNs (36.4% ≥7 mm, 30.8% <7 mm, 24.4% no LLN; *p* = 0.021), but this was not significant in multivariable analyses (HR 1.270; 95% CI, 0.881–1.830; *p* = 0.395). Four-year DM rates did not significantly differ between internal iliac and obturator nodes (24.6% vs 35.5%; *p* = 0.076), respectively. Of the 23 patients who developed LLRs, 16 developed DM (30.4%). Four-year OS was not influenced by the presence of enlarged LLNs versus smaller or no nodes (71.1%, 79.4%, 78.3%; *p* = 0.071; see Appendices 5 at https://links.lww.com/DCR/C206 and 6 at https://links.lww.com/DCR/C207]).

### Internal Iliac Versus Obturator LLNs

Fifty-eight patients had their largest LLN in the internal iliac compartment (20.4%) and 226 in the obturator compartment (79.6%). Four-year LR rates were 9.2% and 18.2% (*p* = 0.211) and LLR rates were 3.6% and 10.3% (*p* = 0.288) for internal iliac and obturator LLNs, respectively.

Enlarged (≥7 mm) internal iliac LLNs (n = 32) and obturator LLNs (n = 90) had mean SA diameters of 9.8 mm (SD 3.2) and 9.2 mm (SD 2.9; *p* = 0.192), respectively. Four-year LR rates for enlarged LLNs were 13.8% for the internal iliac compartment and 23.2% for the obturator compartment (*p* = 0.310), with 4-year LLR rates of 6.6% and 17.7%, respectively (*p* = 0.226; see Appendix 7 at https://links.lww.com/DCR/C205).

### Restaging MRI

In total, 77 of 90 enlarged obturator LLNs (85.6%) and 30 of 32 enlarged internal iliac LLNs (93.8%) underwent restaging MRI after neoadjuvant treatment. Nineteen internal iliac LLNs (19/30, 63%) remained >4 mm on the restaging MRI and resulted in 4-year LR and LLR rates of 22.6% and 11.1%, respectively, compared to 0% and 0% when shrunk to ≤4 mm (*p* = 0.127, *p* = 0.273). For enlarged obturator LLNs that remained >6 mm (n = 32), compared to ≤6 mm (n = 45), 4-year LR rates were 44.5% and 12.8% (*p* = 0.003), and 4-year LLR rates 28.9% and 15.0% (*p* = 0.406), respectively. Obturator nodes, which downsized to ≤4 mm (n = 18), still had a 4-year LLR rate of 18.9%, and for 6 patients in whom LLNs fully disappeared on restaging MRI, 2 developed LLR.

### Malignant Features

At least 1 malignant feature was present in 157 patients with visible internal iliac/obturator LLNs (157/284; 55.3%). The presence of malignant features, regardless of LLN size or location, was associated with increased 4-year LR (20.3% vs 11.3%; *p* = 0.126) and LLR (12.9% vs 3.6%; *p* = 0.024) rates versus those without.

Ninety-eight patients with enlarged LLNs had at least 1 malignant feature present (98/122; 80.3%). Enlarged LLNs with malignant features resulted in higher 4-year LR (23.4% vs 9.1%; *p* = 0.196) and LLR (17.0% vs 5.6%; *p* = 0.189) rates compared to those without malignant features.

Of the 91 patients with intermediate LLNs (SA 5.0–6.9 mm), 43 (47.3%) had malignant features present. Higher 4-year LR and LLR rates were found for these intermediate LLNs with malignant features compared to those without malignant features (LR 17.5% vs 11.4%; *p* = 0.648; LLR 8.2% vs 2.1%; *p* = 0.561). Patients with small LLNs (SA <5 mm) had similar LR (6.7% vs 10.2%; *p* = 0.716) and LLR rates (0% vs 4.4%; *p* = 0.735) in the presence or absence of malignant features, respectively.

## DISCUSSION

This national, cross-sectional study included 1109 patients with cT3-4 rectal cancer located ≤8 cm from the anorectal junction and incorporated training with lateral compartment standardization to provide novel results for the prognostic impact of LLNs. The presence of LLNs, regardless of other characteristics, was associated with a 4-year LLR rate of 8.2%, which increased to 14.3% for enlarged (≥7 mm) external, internal iliac, or obturator LLNs. These outcomes largely verify the Consortium study, which found that enlarged LLNs were associated with 5-year LLR rates of 19.5%.^5^ In contrast, different results were found regarding anatomical location and restaging LLN size, indicating the importance of primarily enlarged LLNs for prognosis and treatment planning. Enlarged LLNs were also associated with a higher risk of DM, but this association was not statistically significant after correcting for primary tumor and margin characteristics.

The definition of lateral nodal disease is important when comparing studies. For example, external iliac nodes were included in the Consortium study, which found a 5-year LLR rate of 19.5%.^5,6^ However, that study and the current study showed that isolated external iliac nodes did not result in LLR, meaning that the LLR rates for obturator/internal iliac nodes were even higher. Other studies do not specify whether external iliac nodes were excluded.^11–15^ Similarly, isolated stretched-out nodes were confirmed to be “benign” in both studies, supporting their exclusion from further analyses.

Anatomical location was noteworthy, with almost 7% 4-year LLR rates for enlarged internal iliac LLNs compared to more than 17% for enlarged obturator LLNs. Recurrence rates for internal iliac LLNs were lower than in the Consortium study and cannot purely be explained by LLN size, as mean SA diameters were not hugely different (9.8 vs 11.7 mm). Two other factors may explain these differences. First, mandatory training with detailed explanations regarding the anatomical classification of LLNs for participating radiologists has likely influenced the categorization of LLNs. This may have led to a stricter interpretation of the lateral borders, meaning that fewer LLNs may have been considered as internal iliac LLNs. In clinical practice, the internal iliac area is usually proportionally narrower than shown in the color atlas of Ogura et al when adhering to the lateral border of the main trunk of the internal iliac artery. This atlas was the only guideline provided in the Consortium study, and radiologists may have relied more on the “color” in the atlas than following the internal iliac main trunk on MRI. Therefore, LLNs located in the transition area may have been defined as internal iliac in the Consortium but as obturator LLNs in the current study. Second, due to the national design, there were fewer LLNs in total (34% vs 58% in the Consortium study), with only 58 internal iliac (20%) and 226 (80%) obturator LLNs, compared to 198 internal iliac (31%) and 448 obturator LLNs (69%) in the Consortium study. Overall, the classification of LLNs into separate anatomical compartments remains challenging. Even after dedicated training, consensus rates among 53 Dutch radiologists for determining LLN location ranged from 75% to 85%.^9^ The current findings and the Consortium study suggest that both compartments can contain aggressive LLNs and that we should predominantly consider primary size in combination with malignant features for clinical suspiciousness.

Important patterns were deduced for the presence of malignant features. Intermediate (5–7 mm) LLNs with at least 1 malignant feature had higher LR and LLR rates (LR 17.5% and LLR 8.2%) compared to those without malignant features (LR 11.4% and LLR 2.1%), respectively. Just as mesorectal nodes are currently classified according to a combination of size and malignant features,^16–21^ upcoming research may indicate a similar possibility for LLNs.^7^ In addition, the importance of restaging sizes could not be confirmed by this study. Although potentially due to limited group numbers, a 15% LLR rate was found for patients with obturator LLNs that decreased in size (≤6 mm), and 29% when remaining >6 mm. Japanese traditions have favored basing treatment decisions on the primary LLN size,^11,12,15^ and the current results appear to support this. Considering that additional LLN dissection (LLND) might be indicated for patients with primarily enlarged LLNs, an important next step is to ascertain whether LLNs received proper irradiation doses. Our research group is examining the irradiation doses received by patients with LLNs ≥5 mm and the outcomes after LLN surgery (not LLND; separate articles).^10,22^ We hypothesize that the majority of LLNs received an adequate dose, meaning that surgical treatment might be imperative to improve oncological outcomes for this population in the future.

The therapeutic implications of this study suggest that patients with primarily enlarged internal iliac and/or obturator LLNs should be treated as suspicious, with 4-year LLR rates of almost 15%. Patients without LLNs displayed rates of around 5%, implying the tangible implications of LLNs. Although enlarged LLNs mainly occur in low, advanced cases, it is important to realize that only one-third of these metastasize in the future, so the majority can be treated curatively, avoiding the morbidity of LLR. Furthermore, there may be a role of LLND in preventing DM.^23^

There are several limitations. The total number of (enlarged) LLNs was limited, meaning that certain features were challenging to examine, which require further exploration in extended data sets. The low number of LLRs meant that multivariable analysis was not possible. Furthermore, the retrospective design means that some data were missing, although thorough verification processes limited this as much as possible. Radiologists were not blinded to the outcome of recurrence, which may have impacted their revision, and finally, interphysician variability during the MRI review process was inevitable, although this was tackled by mandatory training, an extra webinar, and 2 visual atlases.

## CONCLUSION

This national, cross-sectional study of 1109 patients with low, cT3–4 rectal cancer from 60 Dutch hospitals in 2016 with standardized MRI assessment after dedicated training displayed high 4-year ipsilateral LR rates when LLNs were present, with even higher recurrence rates for patients with ≥7 mm LLNs. The presence of (enlarged) LLNs was a significant predictor of LR in multivariable analysis. The results provide a realistic impression of the significance of LLNs at a population level and advocate for the careful consideration of LLNs during clinical practice.

## COLLABORATORS

**Dutch Snapshot Research Group:** Arend G. J. Aalbers, Susanna M. van Aalten, Femke J. Amelung, Marjolein Ankersmit, Imogeen E. Antonisse, Jesse F. Ashruf, Tjeerd S. Aukema, Henk Avenarius, Renu R. Bahadoer, Frans C. H. Bakers, Ilsalien S. Bakker, Fleur Bangert, Renée M. Barendse, Heleen M.D. Beekhuis, Geerard L. Beets, Willem A. Bemelman, Maaike Berbée, Shira H. de Bie, Robert H. C. Bisschops, Robin D. Blok, Liselotte W. van Bockel, Anniek H. Boer, Frank C. den Boer, Evert-Jan G. Boerma, Leonora S. F. Boogerd, Jaap Borstlap, Wernard A. A. Borstlap, Johanna E. Bouwman, Sicco J. Braak, Manon N. G. J. A. Braat, Jennifer Bradshaw, Amarins T. A. Brandsma, Vivian van Breest Smallenburg, Wim T. van den Broek, Sjirk W. van der Burg, Jacobus W. A. Burger, Thijs A. Burghgraef, David W. G. ten Cate, Heleen M. Ceha, Jeltsje S. Cnossen, Robert R. J. Coebergh van den Braak, Esther C. J. Consten, Maaike Corver, Rogier M. P. H. Crolla, Sam Curutchet, Alette W. Daniëls-Gooszen, Paul H. P. Davids, Emmelie N. Dekker, Jan Willem T. Dekker, Ahmet Demirkiran, Tyche Derksen, Arjen L. Diederik, Anne M. Dinaux, Kemal Dogan, Ilse M. van Dop, Kitty E. Droogh-de Greve, Hanneke M. H. Duijsens, Michalda S. Dunker, Johan Duyck, Eino B. van Duyn, Laurentine S. E. van Egdom, Bram Eijlers, Youssef El-Massoudi, Saskia van Elderen, Anouk M. L. H. Emmen, Marc Engelbrecht, Anne C. van Erp, Jeroen A. van Essen, Hans F. J. Fabry, Thomas Fassaert, Eline A. Feitsma, Shirin S. Feshtali, Bas Frietman, Edgar J. B. Furnée, Anne M. van Geel, Elisabeth D. Geijsen, Anna A. W. van Geloven, Michael F. Gerhards, Hugo Gielkens, Renza A. H. van Gils, Lucas Goense, Marc J. P. M. Govaert, Wilhelmina M. U. van Grevenstein, E. Joline de Groof, Irene de Groot, Robbert J. de Haas, Nadia A. G. Hakkenbrak, Mariska D. den Hartogh, Vera Heesink, Joost T. Heikens, Ellen M. Hendriksen, Sjoerd van den Hoek, Erik J. R. J. van der Hoeven, Christiaan Hoff, Anna Hogewoning, Cornelis R. C. Hogewoning, Stefan Hoogendoorn, Francois van Hoorn, René L. van der Hul, Rieke van Hulst, Farshad Imani, Bas Inberg, Martijn P. W. Intven, Pedro Janssen, Chris E. J. de Jong, Jacoline Jonkers, Daniela Jou-Valencia, Bas Keizers, Stijn H. J. Ketelaers, Eva Knöps, Sebastiaan van Koeverden, Sylvia Kok, Stephanie E. M. Kolderman, Fleur I. de Korte, Robert T. J. Kortekaas, Julie C. Korving, Ingrid M. Koster, Jasenko Krdzalic, Pepijn Krielen, Leonard F. Kroese, Eveline J. T. Krul, Derk H. H. Lahuis, Bas Lamme, An A. G. van Landeghem, Jeroen W. A. Leijtens, Mathilde M. Leseman-Hoogenboom, Manou S. de Lijster, Martijn S. Marsman, Milou. H. Martens, Ilse Masselink, Wout van der Meij, Philip Meijnen, Jarno Melenhorst, Dietrich J. L. de Mey, Julia Moelker-Galuzina, Linda Morsink, Erik J. Mulder, Karin Muller, Gijsbert D. Musters, Joost Nederend, Peter A. Neijenhuis, Lindsey C. F. de Nes, M. Nielen, Jan B.J. van den Nieuwboer, Jonanne F. Nieuwenhuis, Joost Nonner, Bo J. Noordman, Stefi Nordkamp, Pim B. Olthof, Steven J. Oosterling, Daan Ootes, Vera Oppedijk, Pieter Ott, Ida Paulusma, Koen C. M. J. Peeters, Ilona T. A. Pereboom, Jan Peringa, Zoë Pironet, Joost D. J. Plate, Fatih Polat, Ingrid G. M. Poodt, Lisanne A. E. Posma, Jeroen F. Prette, Bareld B. Pultrum, Seyed M. Qaderi, Jan M. van Rees, Rutger-Jan Renger, Anouk J. M. Rombouts, Lodewijk J. Roosen, Ellen A. Roskott-ten Brinke, Joost Rothbarth, Dennis B. Rouw, Tom Rozema, Heidi Rütten, Harm J. T. Rutten, Marit E. van der Sande, Boudewijn E. Schaafsma, Renske A. Schasfoort, Merel M. Scheurkogel, Arjan P. Schouten van der Velden, Wilhelmina H. Schreurs, Puck M. E. Schuivens, Colin Sietses, Petra C. G. Simons, Marjan J. Slob, Gerrit D. Slooter, Martsje van der Sluis, Bo P. Smalbroek, Anke B. Smits, Ernst J. Spillenaar-Bilgen, Patty H. Spruit, Tanja C. Stam, Jaap Stoker, Aaldert K. Talsma, Sofieke J. D. Temmink, G. Y. Mireille The, Jeroen A. W. Tielbeek, Aukje A. J. M. van Tilborg, Fiek van Tilborg, Dorothée van Trier, Jurriaan B. Tuynman, Maxime J. M. van der Valk, Inge J. S. Vanhooymissen, G. Boudewijn C. Vasbinder, Cornelis J. Veeken, Laura A. Velema, Anthony W. H. van de Ven, Emiel G. G. Verdaasdonk, Wouter M. Verduin, Tim Verhagen, Paul M. Verheijen, Maarten Vermaas, An-Sofie E. Verrijssen, Anna V. D. Verschuur, Harmke Verwoerd-van Schaik, Roy F. A. Vliegen, Sophie Voets, F. Jeroen Vogelaar, Clementine L. A. Vogelij, Johanna Vos-Westerman, Marianne de Vries, Joy C. Vroemen, Bas S. T. van Vugt, Johannes A. Wegdam, Bob J. van Wely, Marinke Westerterp, Paul P. van Westerveld, Henderik L. van Westreenen, Allard G. Wijma, Johannes H. W. de Wilt, Bart W. K. de Wit, Fennie Wit, Karlijn Woensdregt, Victor van Woerden, Floor S. W. van der Wolf, Sander van der Wolk, Johannes M. Wybenga, Edwin S. van der Zaag, Bobby Zamaray, Herman J. A. Zandvoort, Dennis van der Zee, Annette Zeilstra, Kang J. Zheng, David D. E. Zimmerman, and Marcel Zorgdrager.

## Supplementary Material
