# Supplementary material for: Prognostic Implications of Lateral Lymph Nodes in Rectal Cancer: A Population-Based Cross-sectional Study With Standardized Radiological Evaluation After Dedicated Training
Source: Dis Colon Rectum. 2023 Jun 1;67(1):42–53. doi: 10.1097/DCR.0000000000002752 (PMC10715698; doi:10.1097/DCR.0000000000002752)
Supplement: Supplementary file 1 [file dcr-67-42-s002.pdf]

Appendix 1: Data management & privacy

The Dutch ColoRectal Audit (DCRA) has all baseline and short-term oncological outcomes registered for patients who were treated for rectal carcinoma in the Netherlands. This Snapshot study expanded the available data in the DCRA for 3107 of the 3178 potentially eligible patients who were treated for primary rectal carcinoma in the Netherlands in 2016.

Data collection consisted of three parts, of which only the first two parts are relevant to the present study. In part 1, the surgical team collected all additional information per patient, such as baseline characteristics, procedural data, and the short- and long-term oncological and surgical outcomes. The local surgical team only had access to data for patients in their center within part 1. Once part 1 was completed, MRDM imported eligible patients to part 2 in a completely separate data collection location. This meant that local collaborators could not access any information about their patients outside their specific part or center. Sixty centers also participated in part 2. In this section, abdominal radiology consultants were asked to re-review all primary and restaging MR-images for the selected patients.

The project data was processed and stored anonymously by Medical Research Data Management (MRDM, Deventer, the Netherlands). MRDM is responsible for the data processing of the DCRA and is NEN7510 and ISO27001 certified.

The central coordinating researchers received fully anonymized data and dates of birth were only provided as a year of birth. All other dates, such as the date of the primary MRI, were provided with a possible 10-day spread, to minimize any risk of breach in privacy. MRI-reports were copied by the local collaborative team into the database anonymously and did not include any patient-specific information.
