# Supplementary figures and images for: Prognostic Implications of Lateral Lymph Nodes in Rectal Cancer: A Population-Based Cross-sectional Study With Standardized Radiological Evaluation After Dedicated Training
Source: Dis Colon Rectum. 2023 Jun 1;67(1):42–53. doi: 10.1097/DCR.0000000000002752 (PMC10715698; doi:10.1097/DCR.0000000000002752)

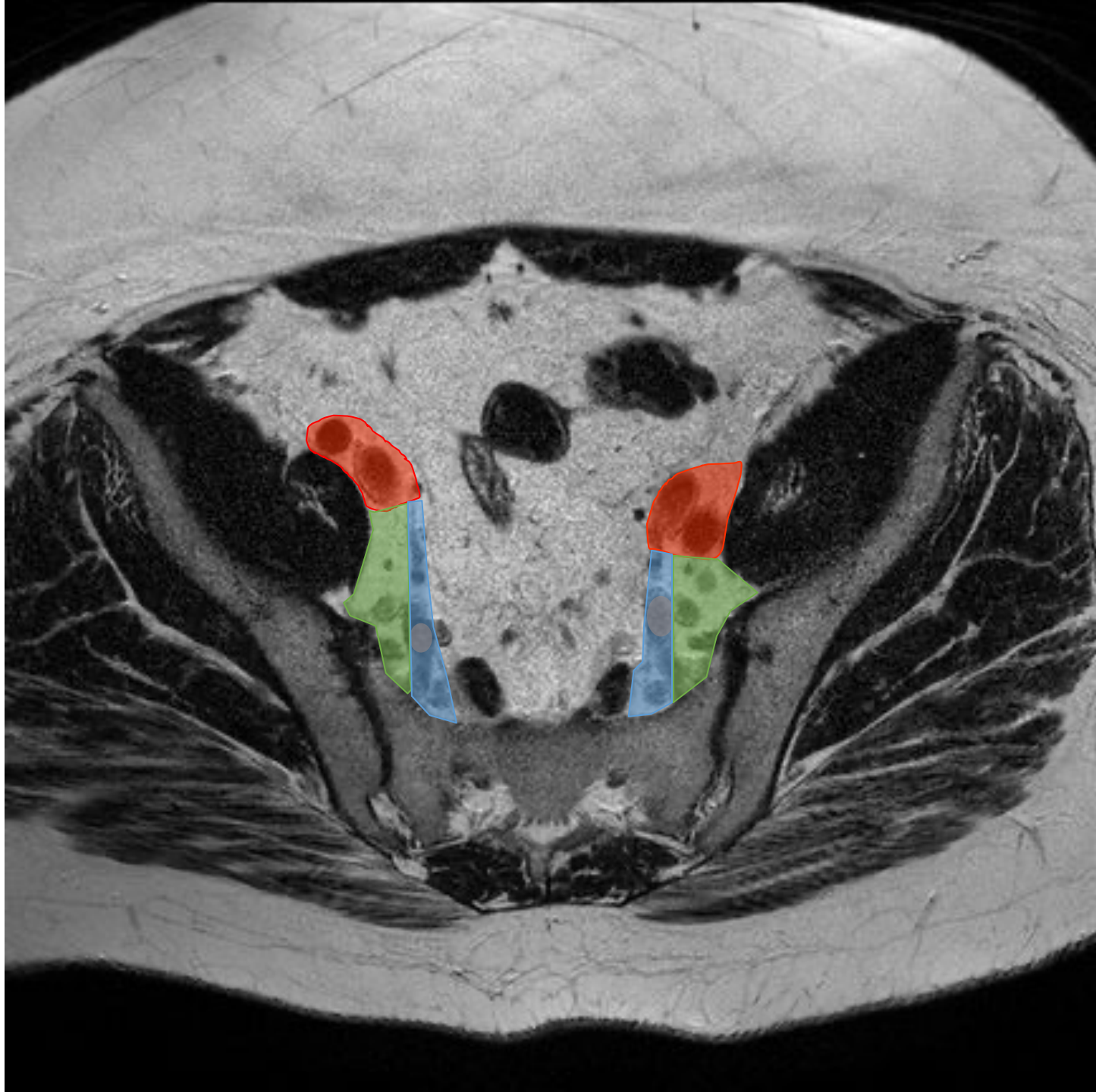

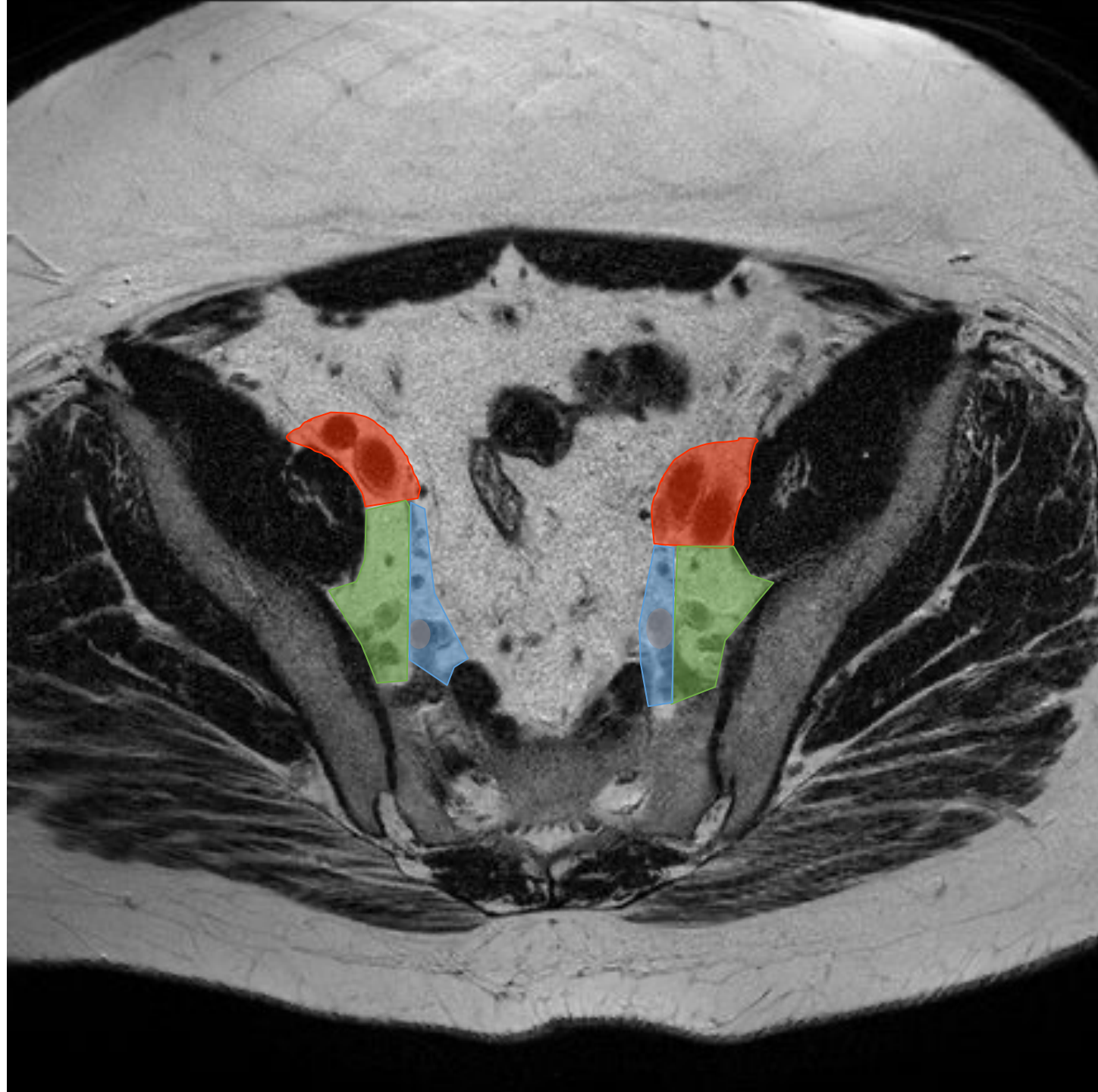

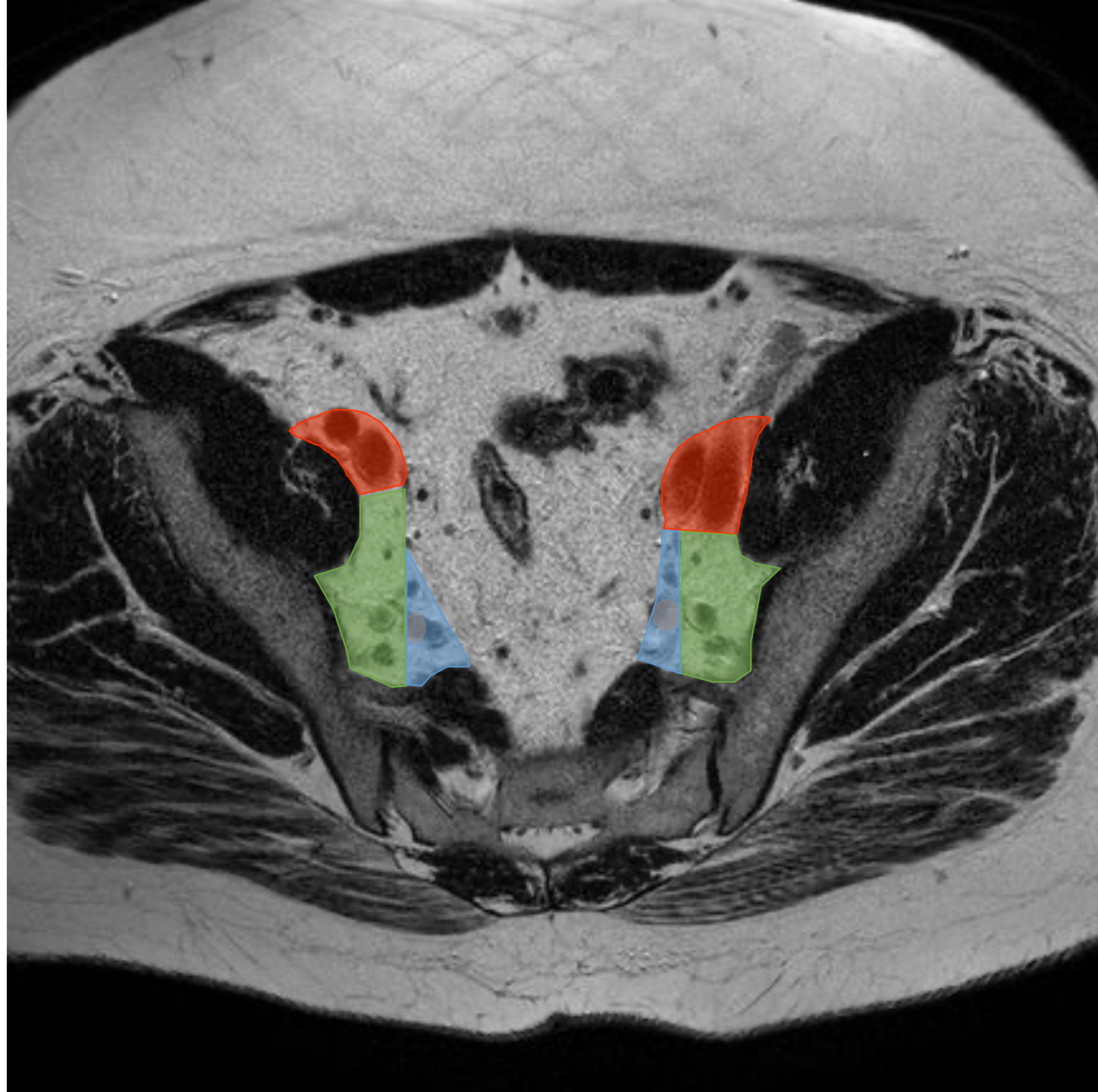

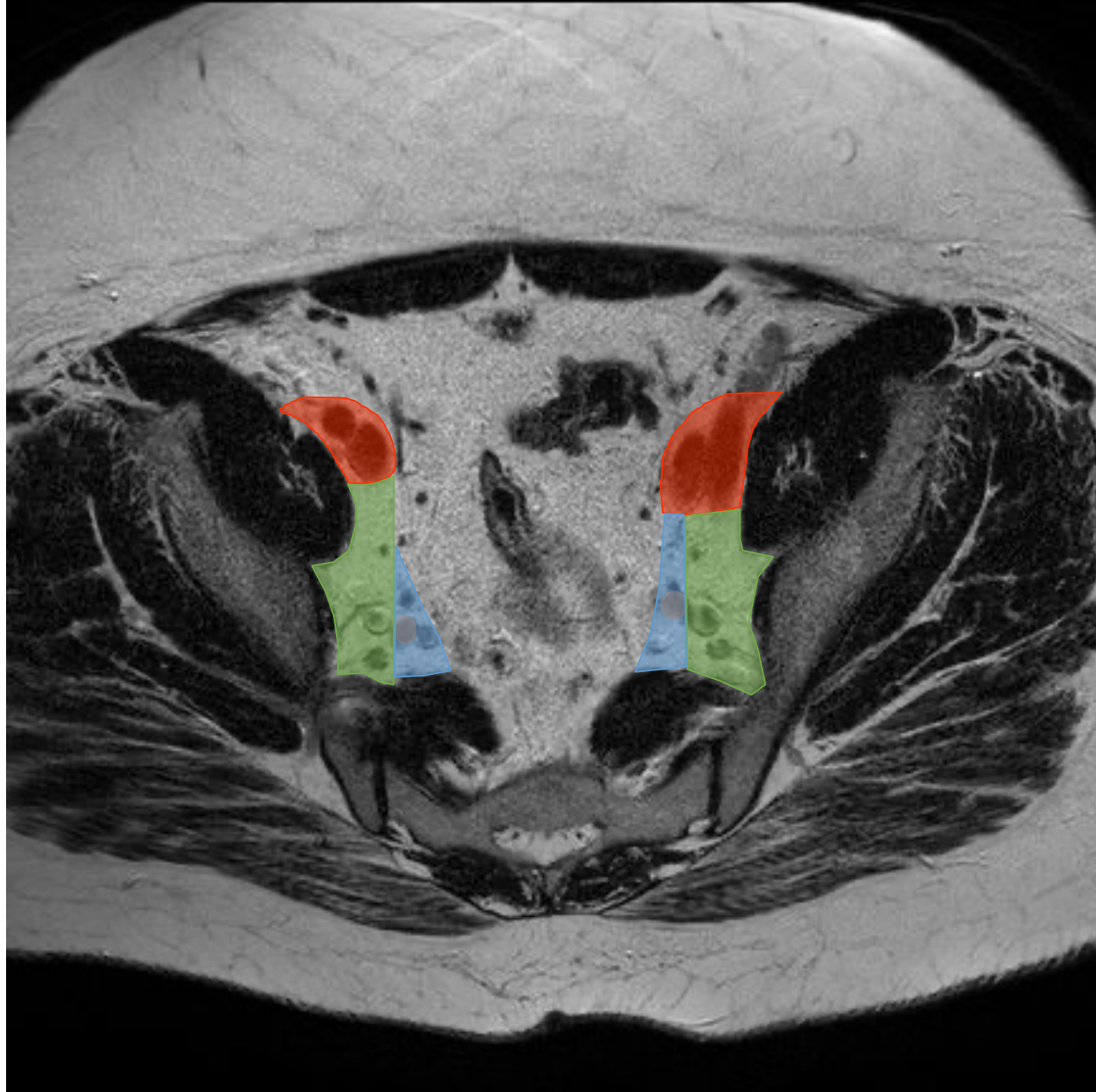

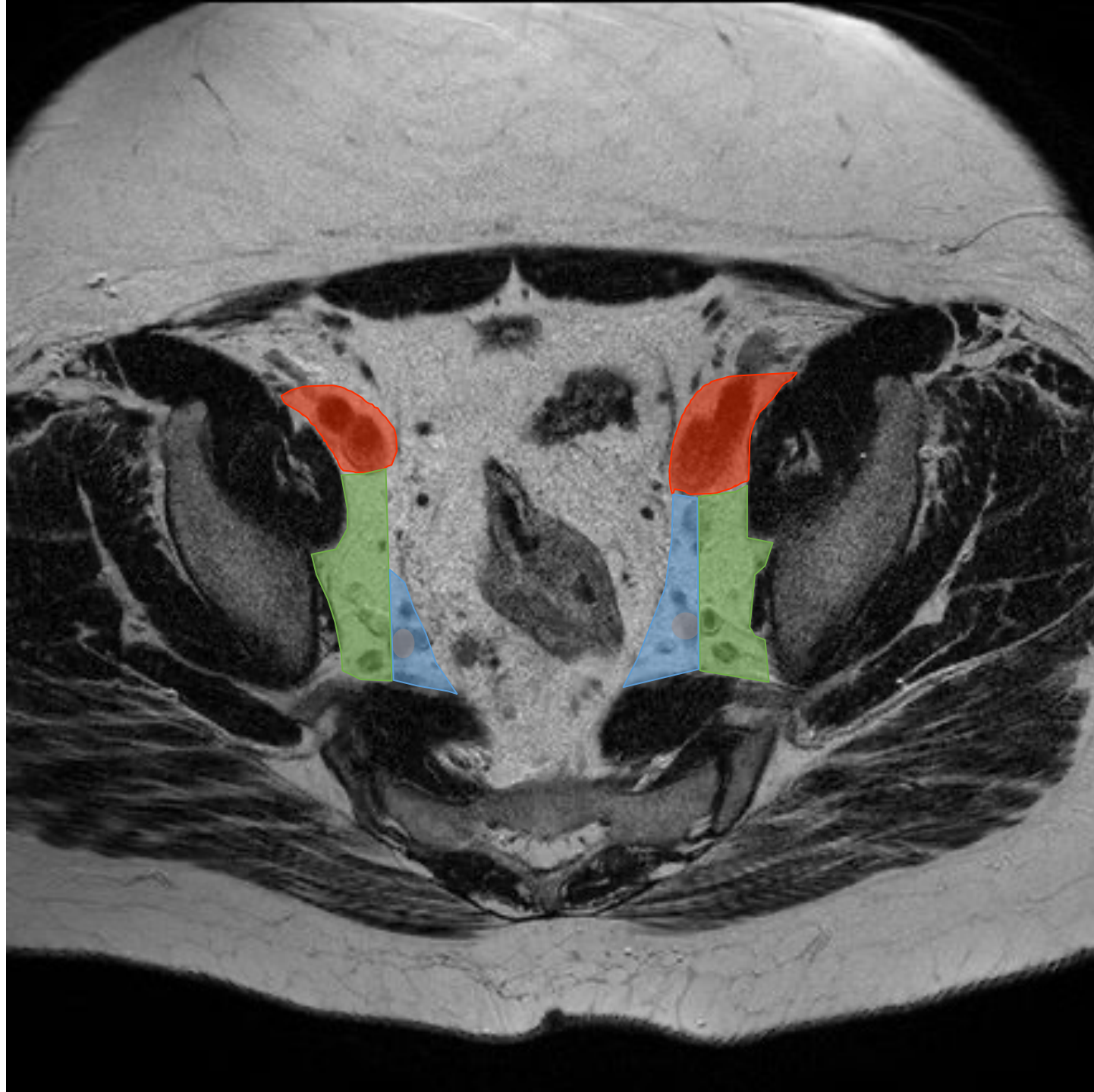

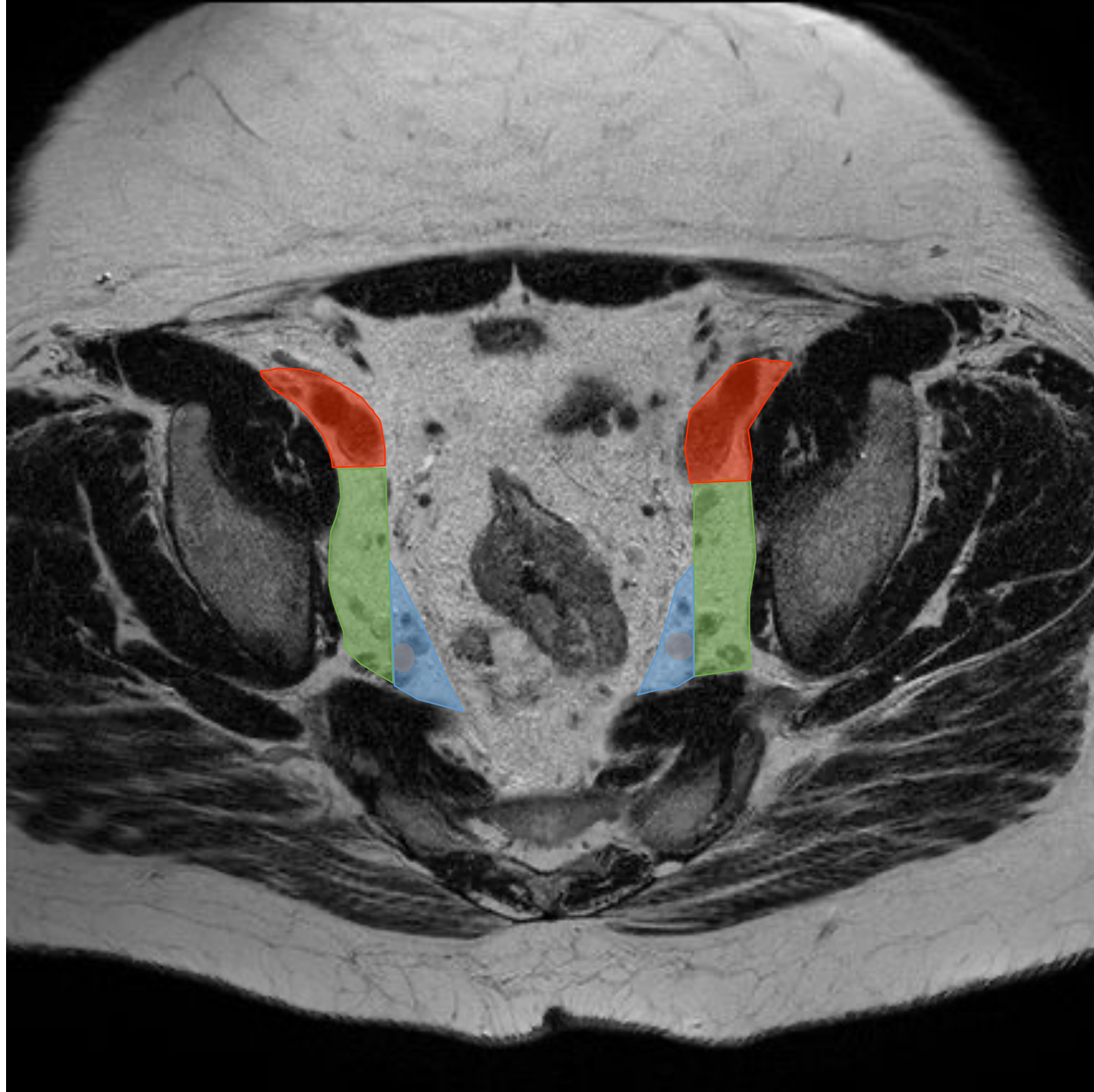

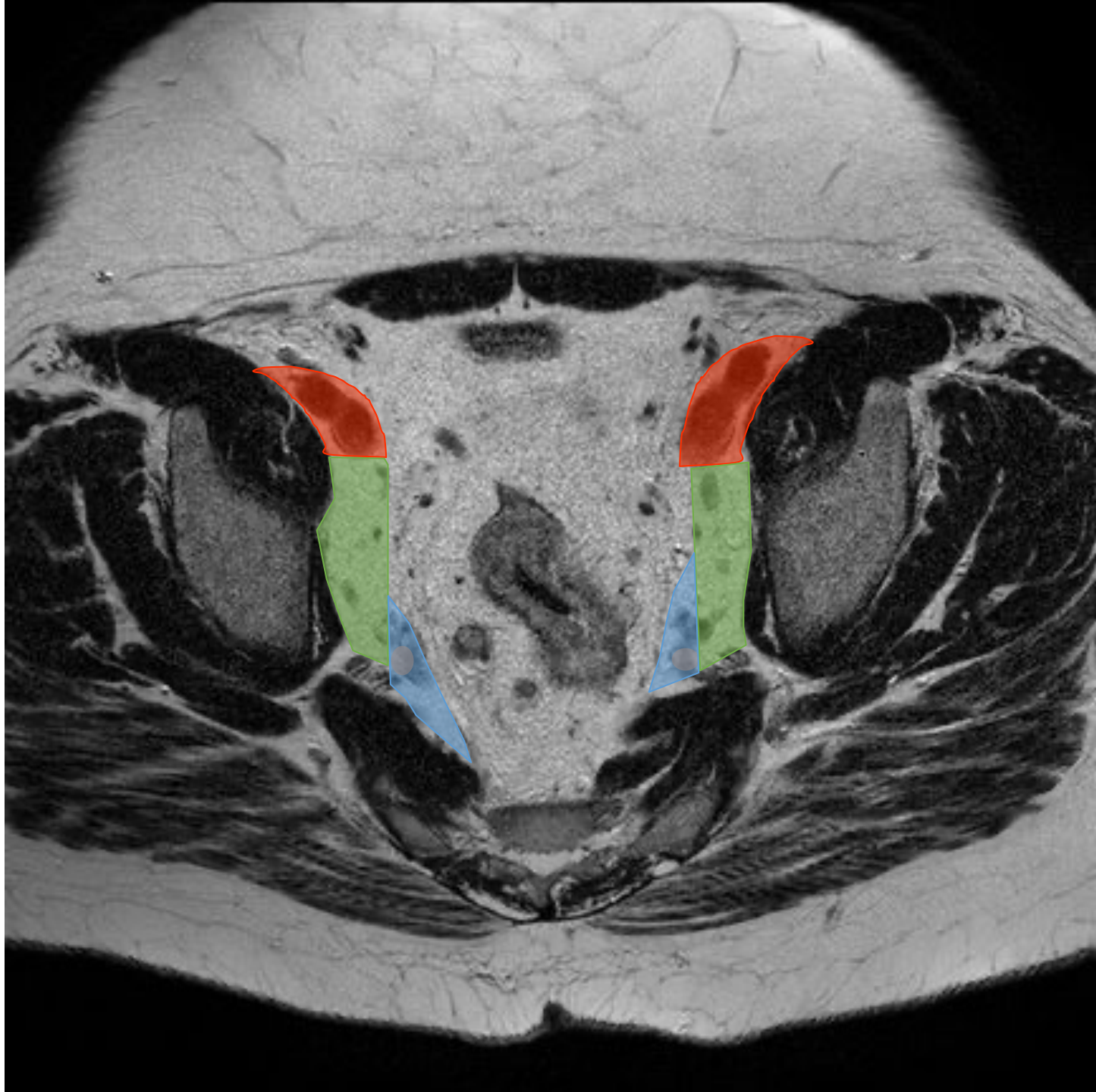

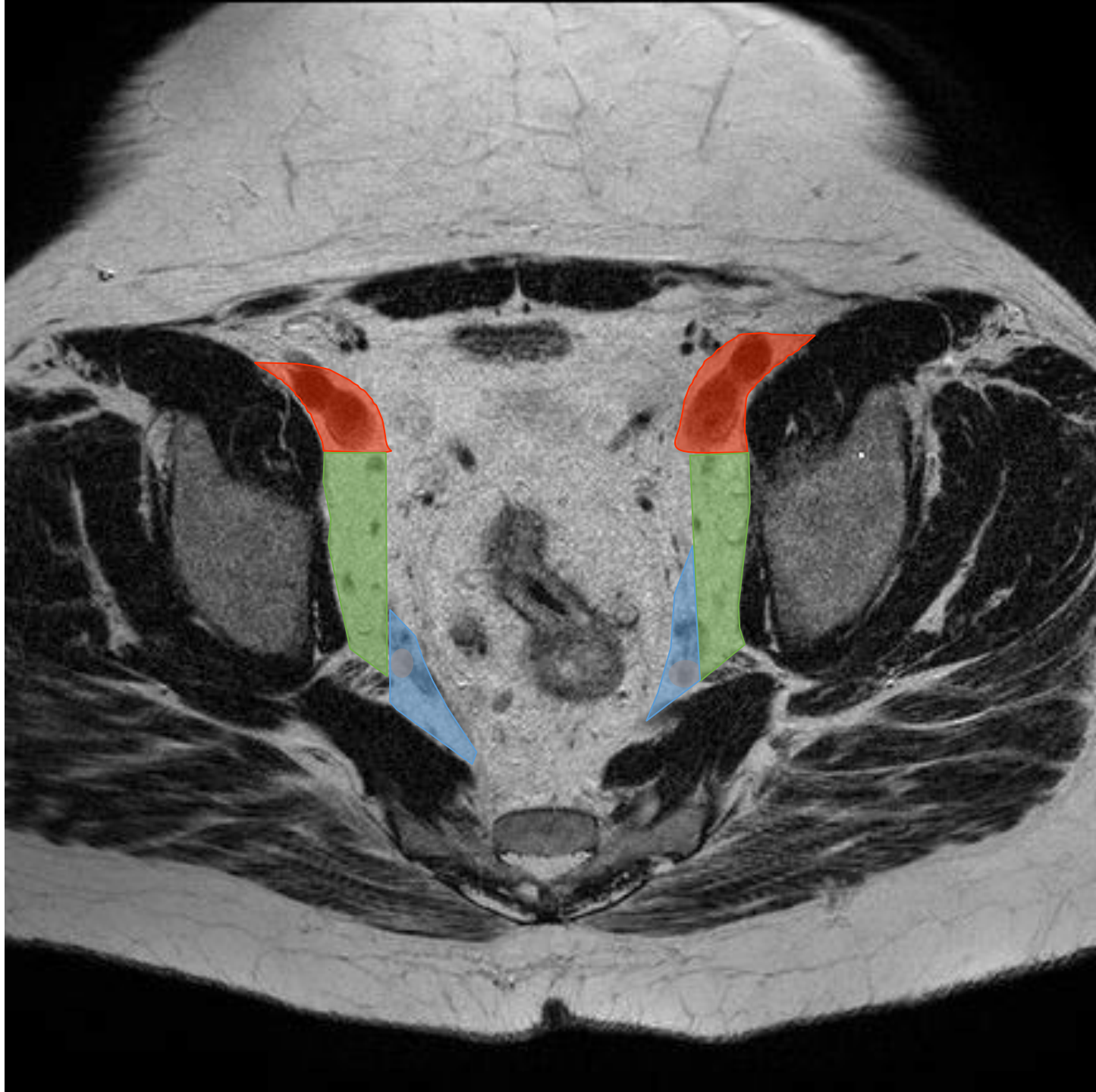

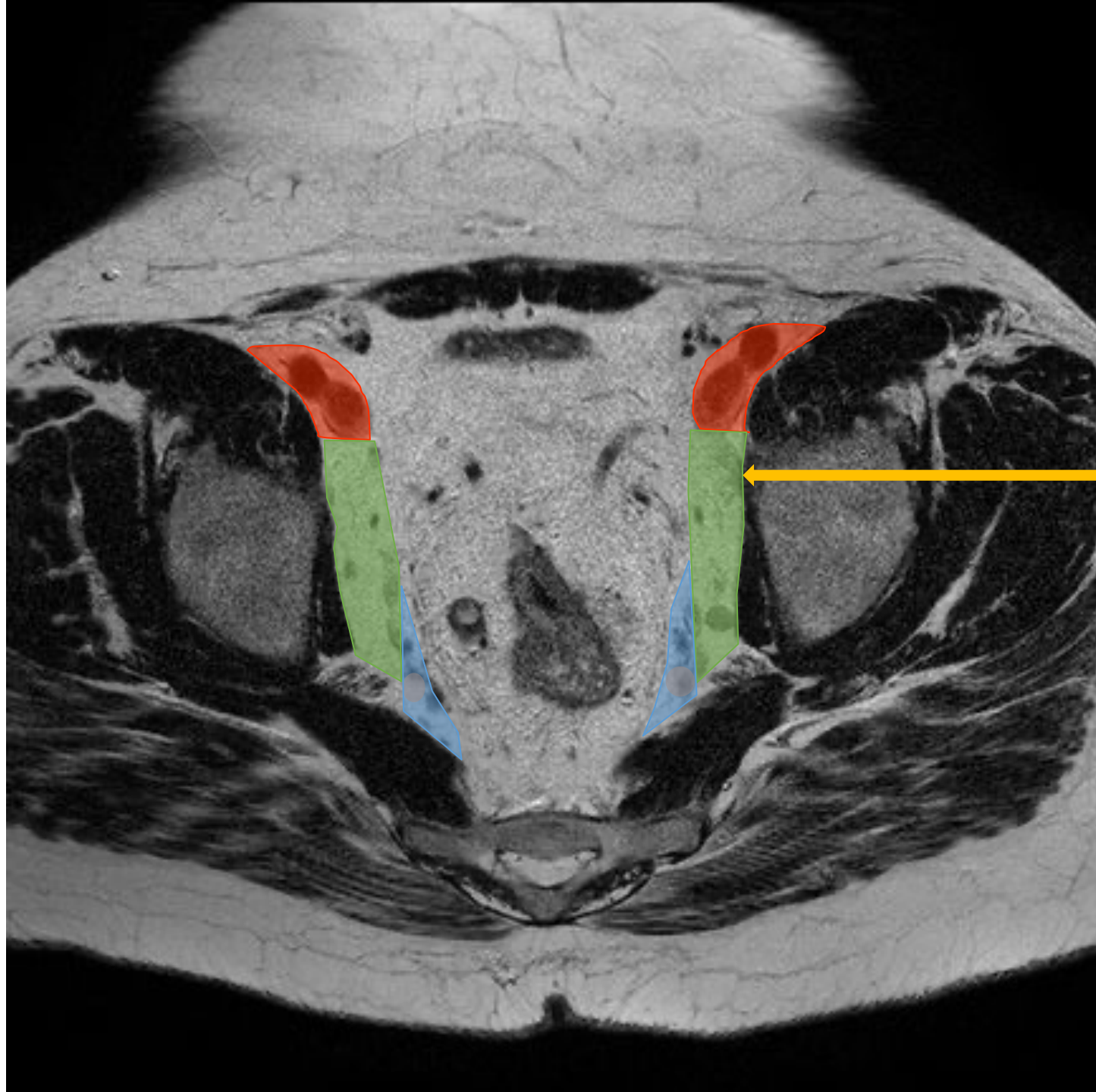

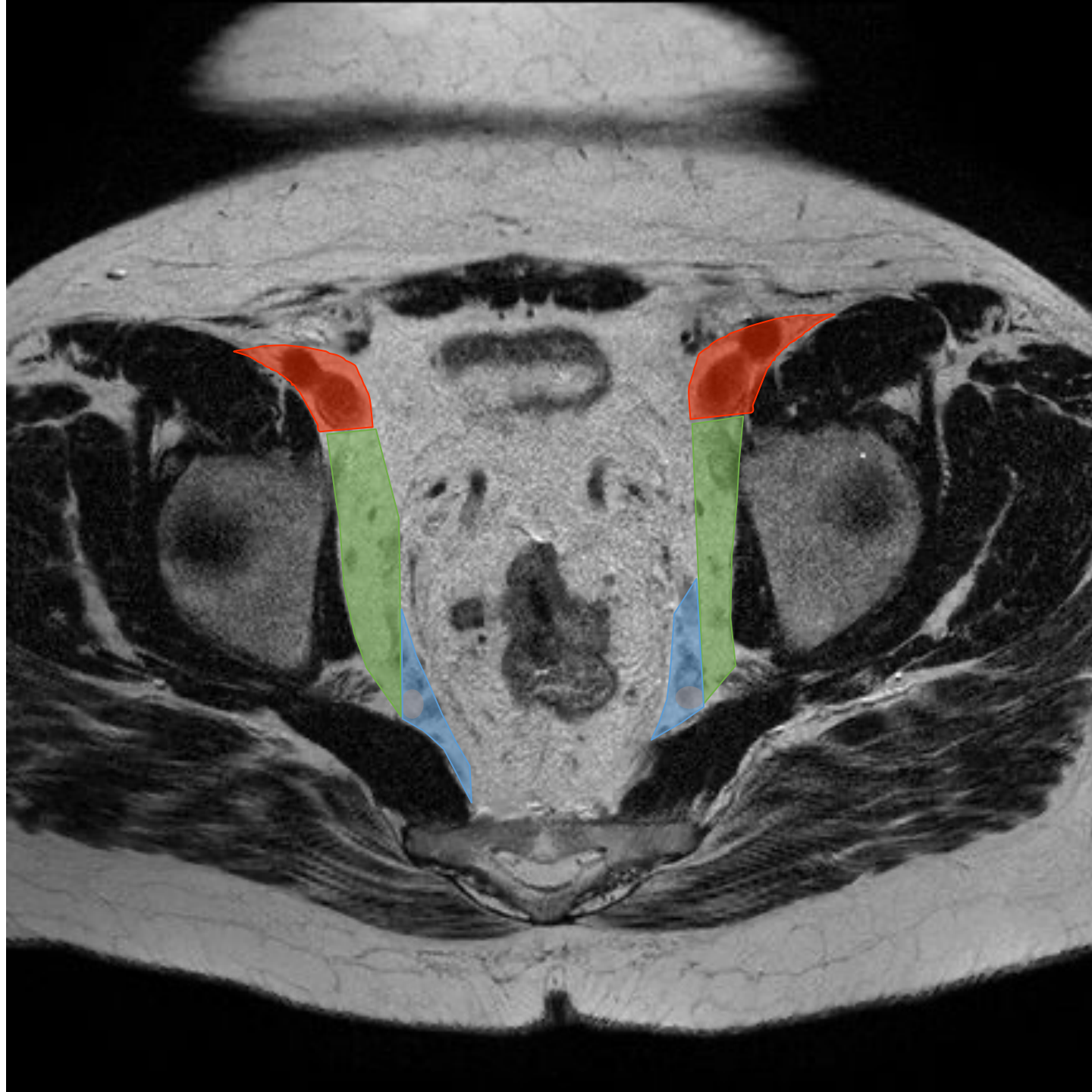

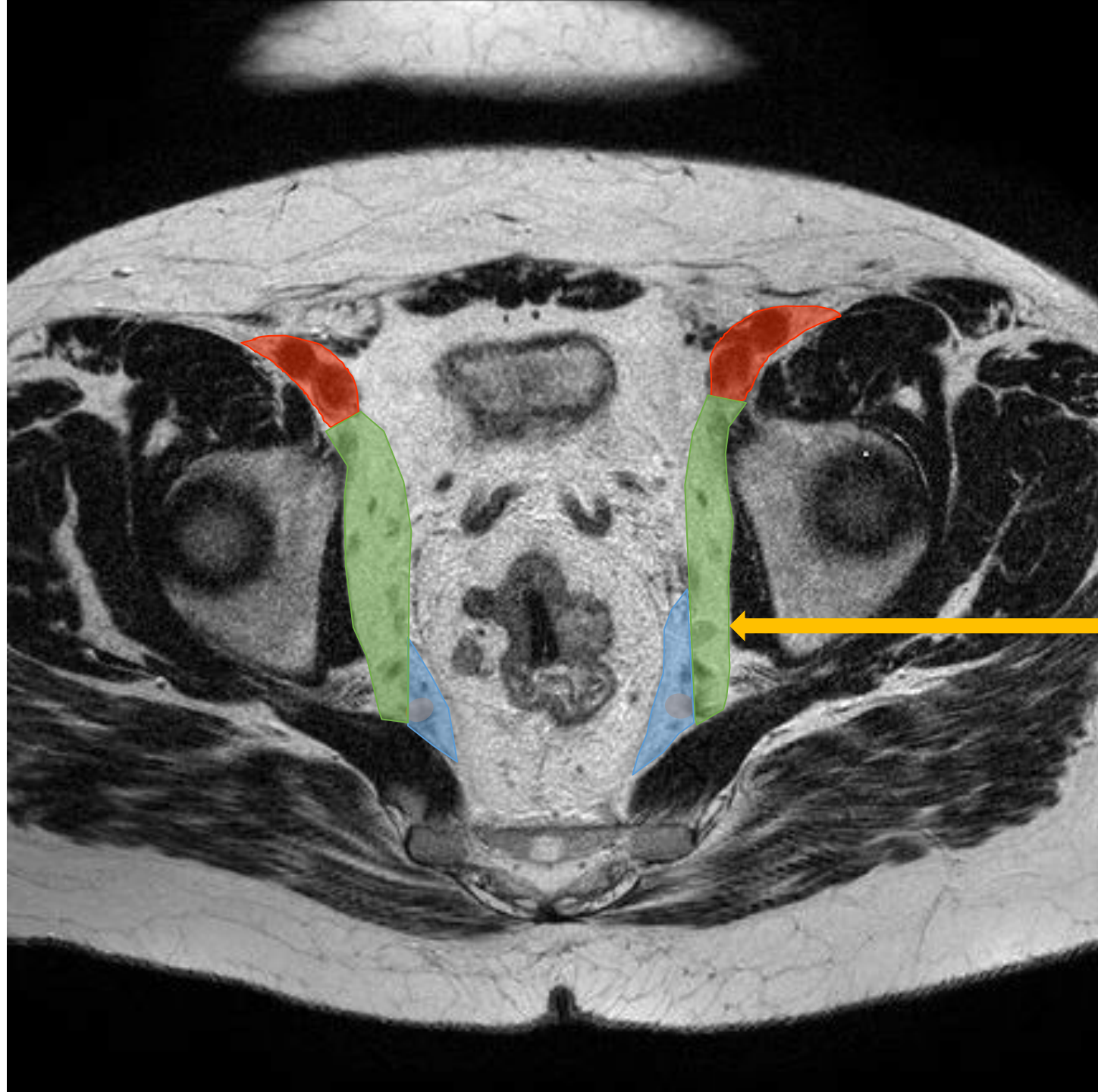

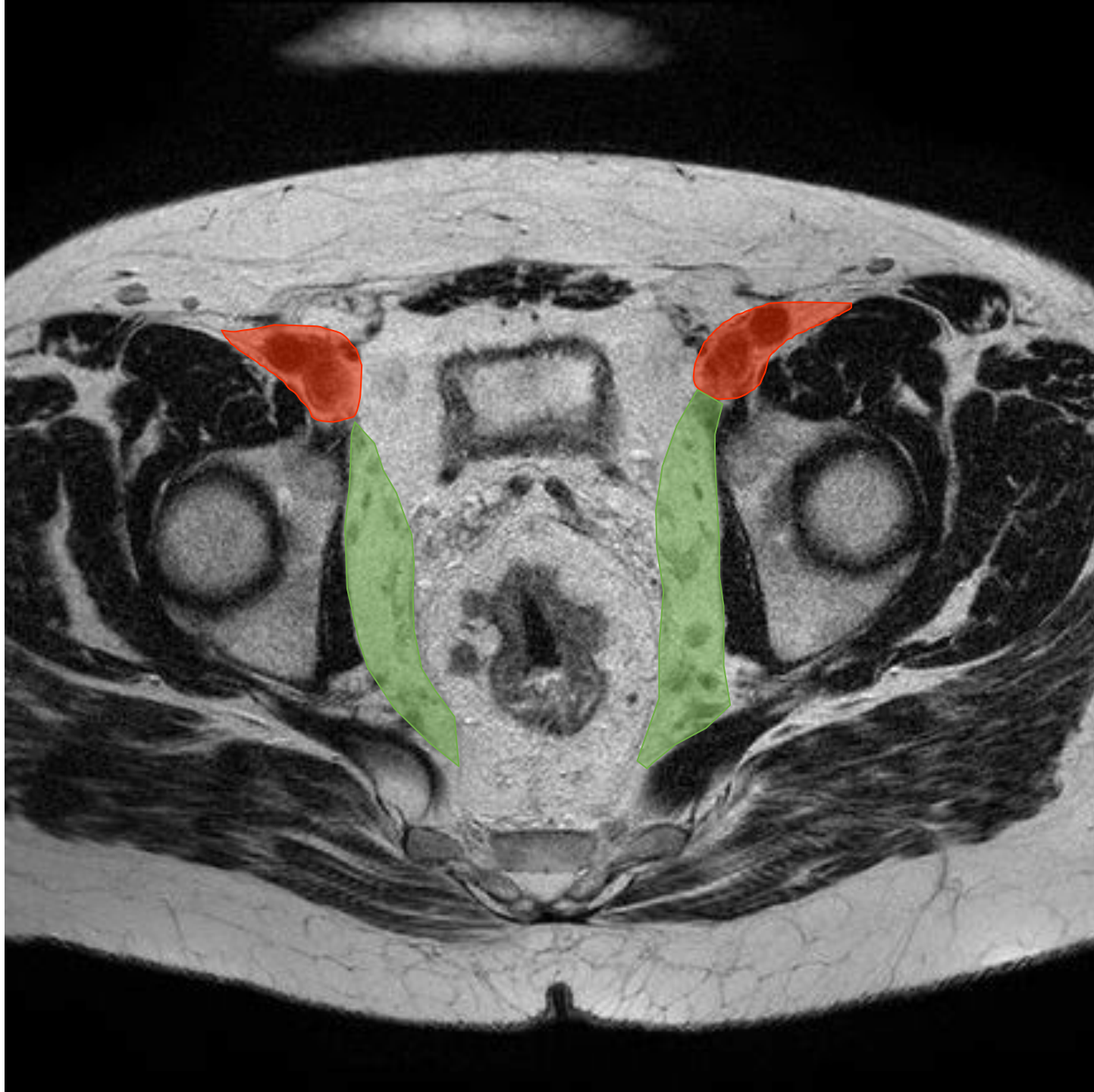

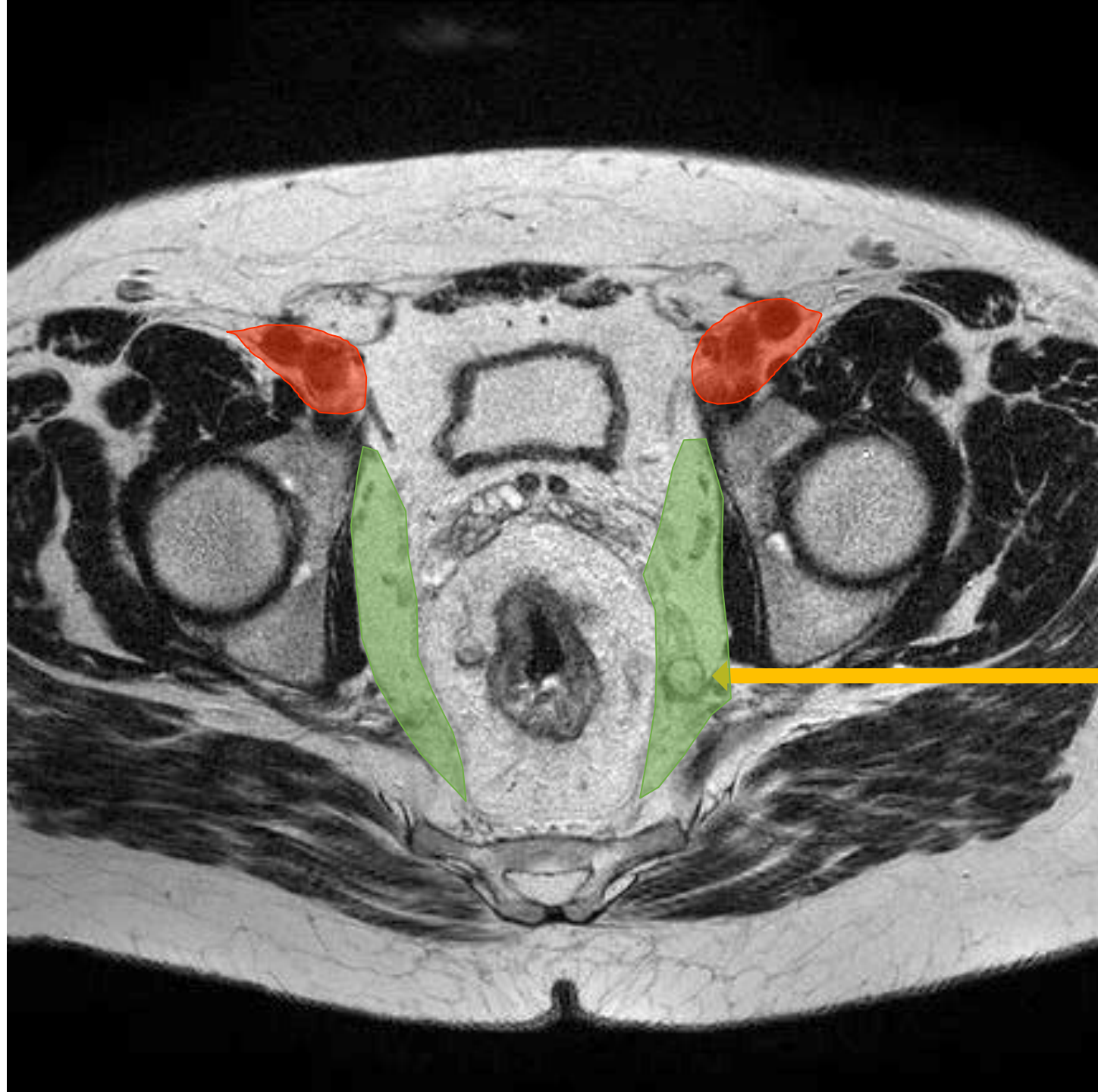

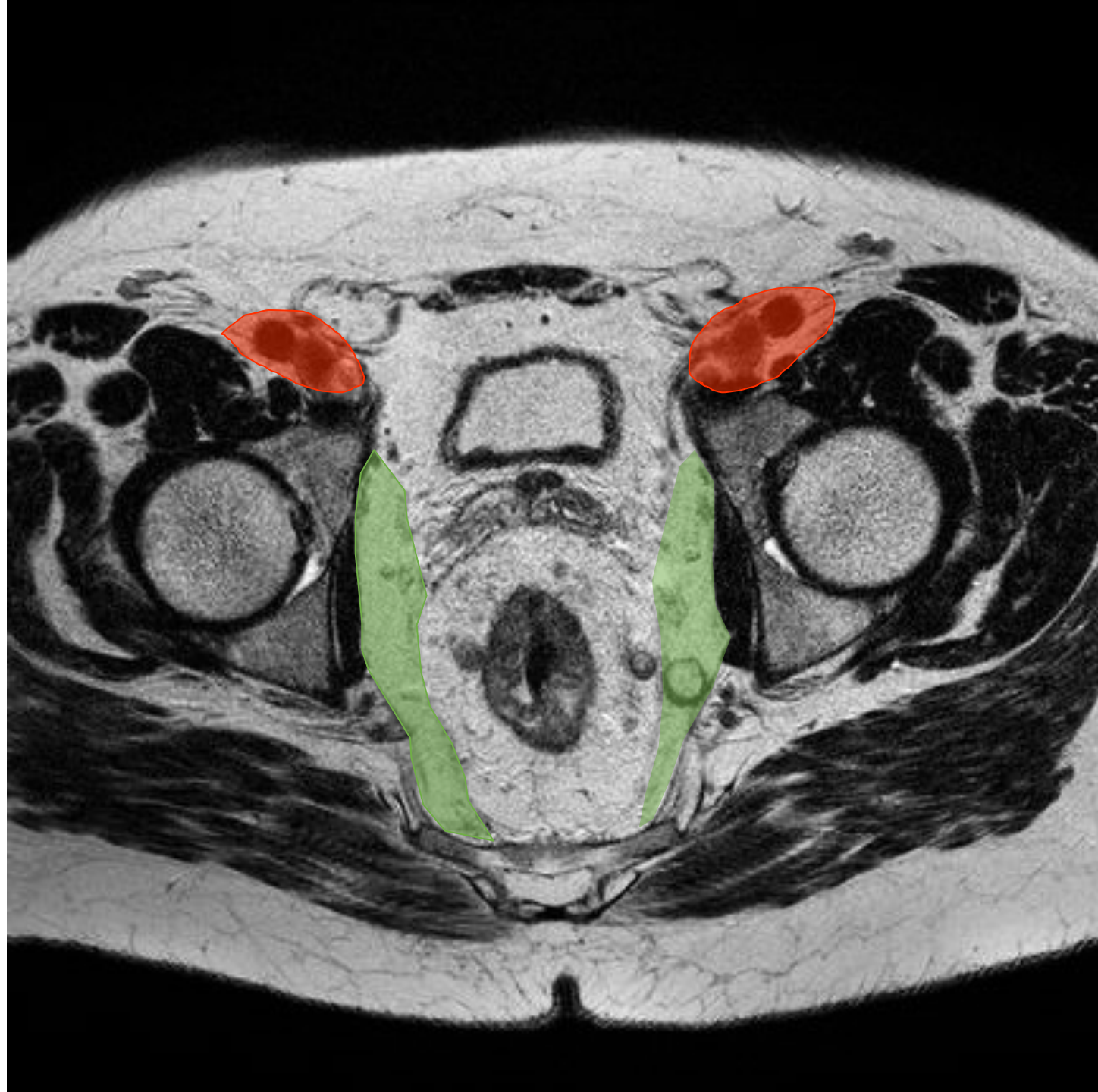

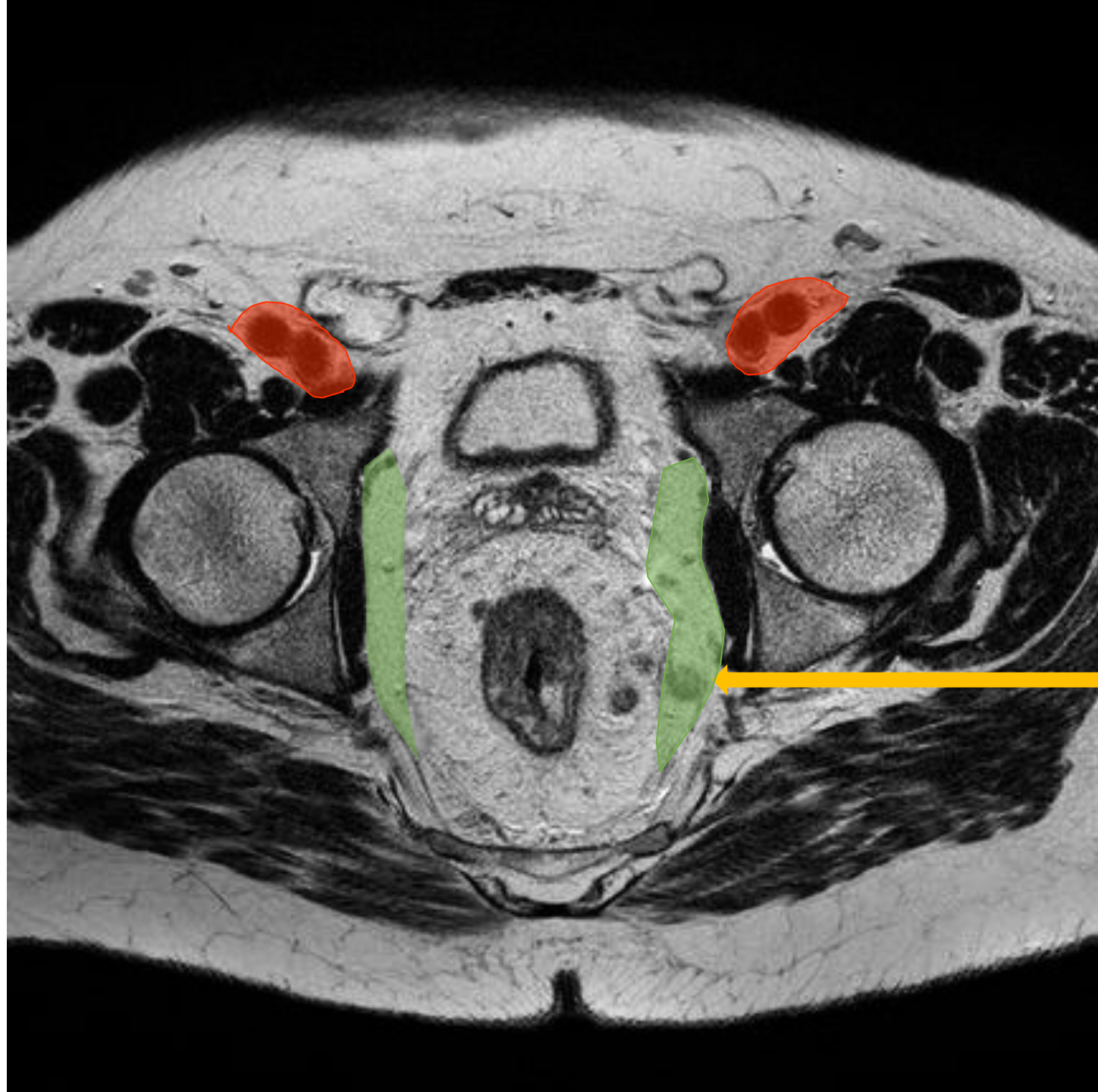

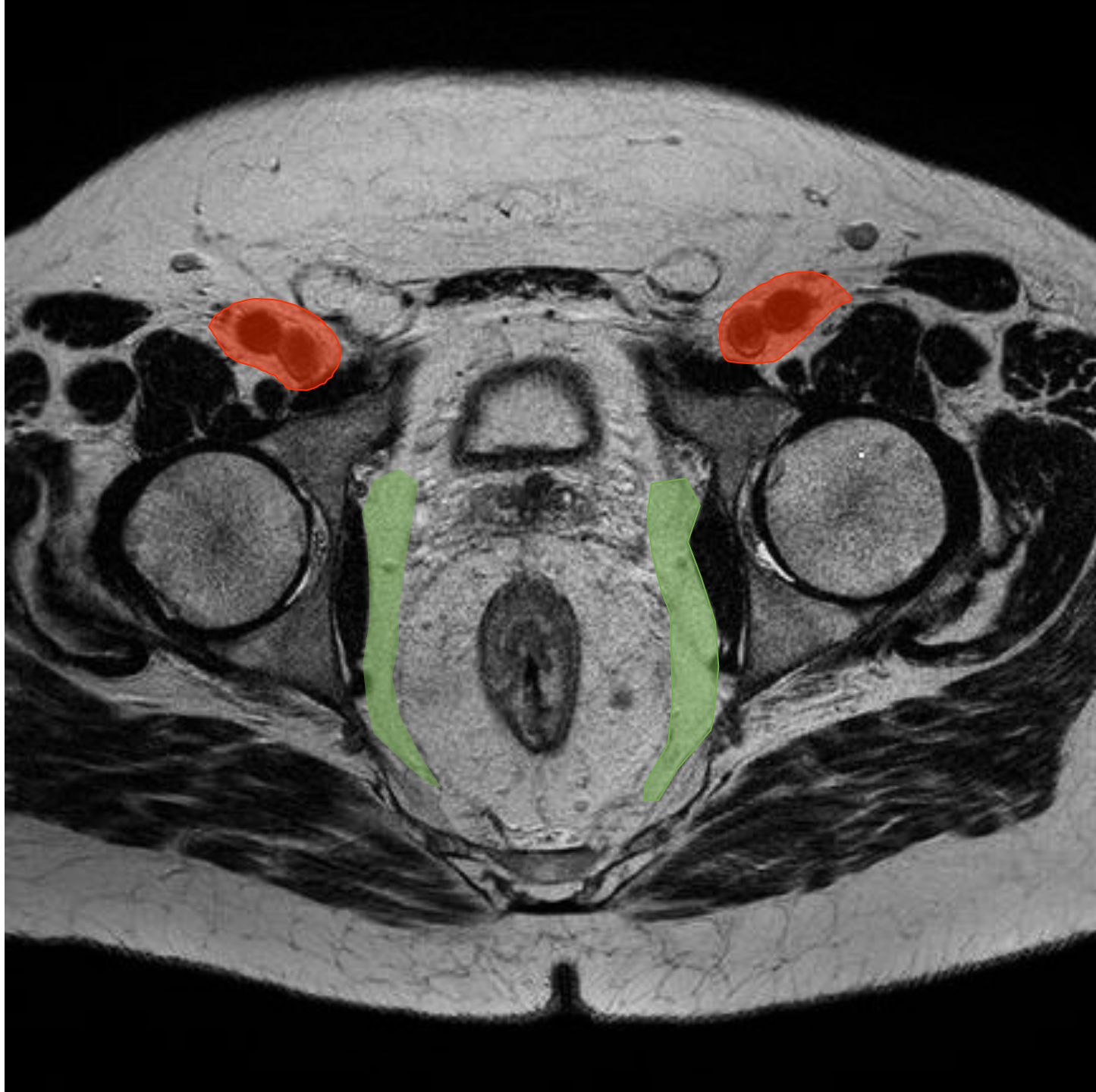

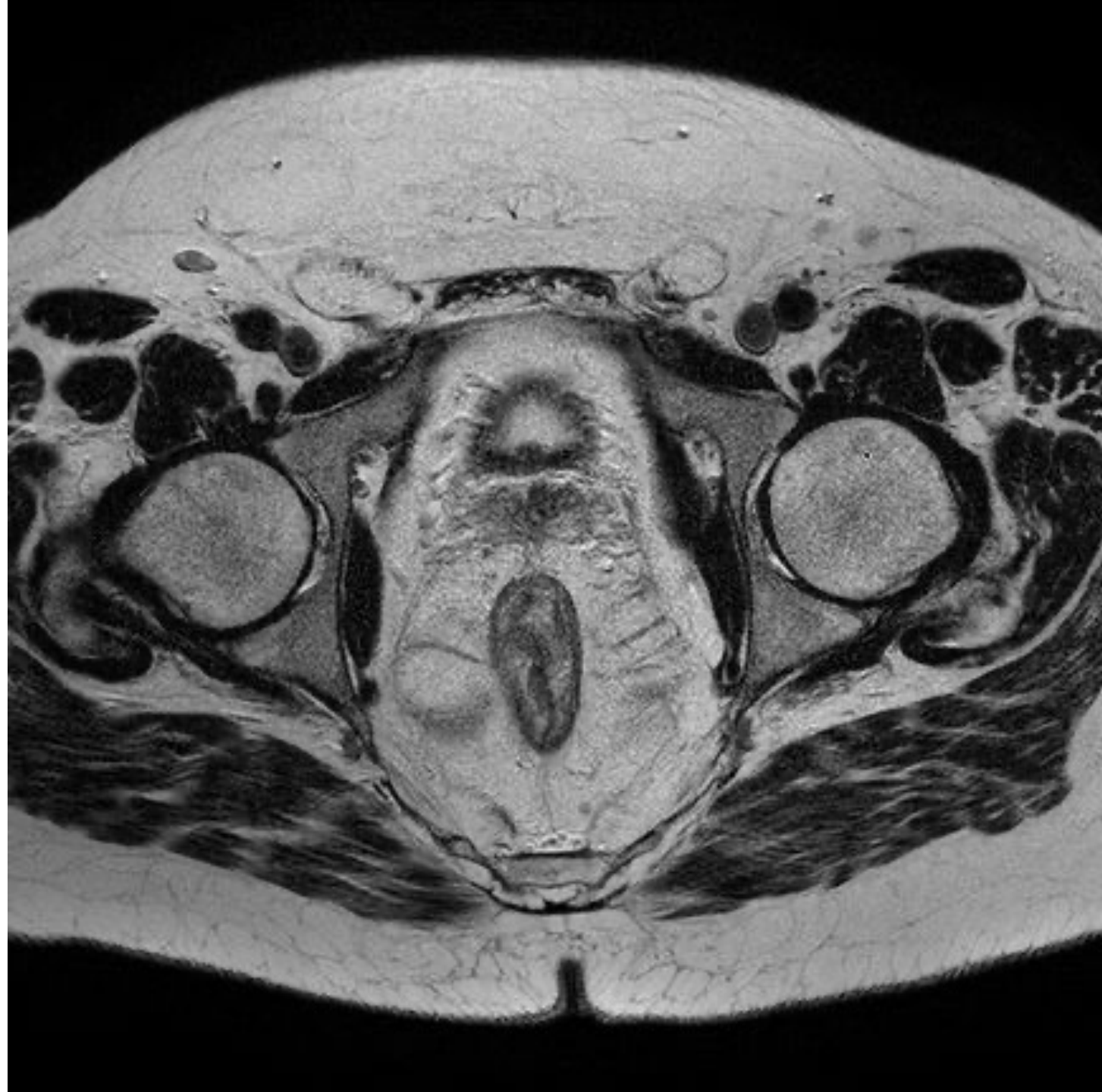

Supplement: Supplementary file 2 [file dcr-67-42-s003.pdf]
