# Supplementary material for: Prognostic Implications of Lateral Lymph Nodes in Rectal Cancer: A Population-Based Cross-sectional Study With Standardized Radiological Evaluation After Dedicated Training
Source: Dis Colon Rectum. 2023 Jun 1;67(1):42–53. doi: 10.1097/DCR.0000000000002752 (PMC10715698; doi:10.1097/DCR.0000000000002752)
Supplement: Supplementary file 3 [file dcr-67-42-s004.pdf]

Appendix 3. 4-year LR and LLR rates for 314 patients with LLNs in different anatomical locations from the total cohort of 890 patients with low, locally advanced rectal cancer who received neoadjuvant therapy.

N=314 patients with LLNs	N (%)	N with LR	4-yr LR, %	N with LLR	4-yr LLR, %
**External iliac LLNs**	17 (5.4)	1	6.2%	0	0
**Obturator LLNs**					
Obturator LLNs	226 (72.0)	36	18.2%	20	10.2%
Stretched-out LLNs*	13 (4.1)	1	11.1%	0	0
**Internal iliac LLNs**	58 (18.5)	6	9.2%	3	3.6%

*subtype of obturator LLNs, defined as follows: long-axis at least twice the length of the short-axis, maximum short-axis diameter of 5mm, no malignant features present and no change/increase in the restaging MRI.
