# Supplementary material for: Prognostic Implications of Lateral Lymph Nodes in Rectal Cancer: A Population-Based Cross-sectional Study With Standardized Radiological Evaluation After Dedicated Training
Source: Dis Colon Rectum. 2023 Jun 1;67(1):42–53. doi: 10.1097/DCR.0000000000002752 (PMC10715698; doi:10.1097/DCR.0000000000002752)
Supplement: Supplementary file 4 [file dcr-67-42-s005.pdf]

Appendix 4: Explorative univariable analysis to determine the most appropriate cut-off value for SA diameter of internal iliac and obturator LLNs in patients (n=284) who received neoadjuvant treatment.

N=284	N (%)	4-yr LR	p-value	N (%)	4-yr LLR	p-value
SA ≥1mm<br>SA <1mm	284 (100)<br>0	16.4%	-	284 (100)<br>0	8.8%	-
SA ≥2mm<br>SA <2mm	284 (100)<br>0	16.4%	-	284 (100)<br>0	8.8%	.
SA ≥3mm<br>SA <3mm	273 (96)<br>11 (4)	16.2%<br>22.2%	.567	273 (96)<br>11 (4)	8.6%<br>11.1%	.722
SA ≥4mm<br>SA <4mm	253 (89)<br>31 (11)	15.7%<br>21.9%	.343	253 (89)<br>31 (11)	9.3%<br>3.8%	.791
SA ≥5mm<br>SA <5mm	213 (75)<br>71 (25)	18.0%<br>10.9%	.256	213 (75)<br>71 (25)	10.4%<br>3.4%	.199
SA ≥6mm<br>SA <6mm	168 (59)<br>116 (41)	20.3%<br>10.5%	.107	168 (59)<br>116 (41)	11.8%<br>4.2%	.147
SA ≥7mm<br>SA <7mm	122 (43)<br>162 (57)	20.8%<br>13.1%	.165	122 (43)<br>162 (57)	14.7%<br>4.4%	**.018**
SA ≥8mm<br>SA <8mm	69 (24)<br>215 (76)	26.8%<br>13.1%	**.020**	69 (24)<br>215 (76)	19.4%<br>5.5%	**.003**
SA ≥9mm<br>SA <9mm	48 (17)<br>236 (83)	33.8%<br>12.9%	**.001**	48 (17)<br>236 (83)	23.6%<br>6.0%	**.001**
