# Supplementary material for: Prognostic Implications of Lateral Lymph Nodes in Rectal Cancer: A Population-Based Cross-sectional Study With Standardized Radiological Evaluation After Dedicated Training
Source: Dis Colon Rectum. 2023 Jun 1;67(1):42–53. doi: 10.1097/DCR.0000000000002752 (PMC10715698; doi:10.1097/DCR.0000000000002752)
Supplement: Supplementary file 5 [file dcr-67-42-s006.pdf]

Appendix 7. Baseline criteria of the 891 patients with cT3-4M0 rectal cancer located ≤8cm from the anorectal junction based on MRI re-review who received neoadjuvant radiotherapy divided into four groups: no LLNs, small (<7mm) LLNs, enlarged (≥7mm) internal iliac LLNs and obturator LLNs.

Primarily enlarged (≥7mm) internal iliac or obturator LLNs versus smaller (<7mm) LLNs or no LLNs	Internal iliac LLNs, N=32 (%)	Obturator LLNs N=90 (%)	<7 mm LLNs N=162 (%)	No LLNs N=607 (%)	*p* value
Gender: male	22 (68.8)	59 (65.6)	107 (66.0)	393 (64.7)	0.966
Age					**0.020**
<55 years	4 (12.5)	10 (11.1)	7 (4.3)	29 (4.8)	
55-74 years	22 (68.8)	46 (51.1)	87 (53.7)	317 (52.2)	
>75 years	6 (18.8)	34 (37.8)	68 (42.0)	261 (43.0)	
Distance of tumor from ARJ					**<0.001**
0.0-4.0cm	22 (68.8)	72 (80.0)	97 (59.9)	338 (55.7)	
4.1-8.0cm	10 (31.3)	18 (20.0)	65 (40.1)	269 (44.3)	
Tumor according to LOREC criteria					0.055
On/below	21 (65.6)	66 (73.3)	99 (61.1)	355 (58.5)	
Above	11 (34.4)	24 (26.7)	63 (38.9)	252 (41.5)	
Clinical T-stage					**<0.001**
T3a (<1mm beyond muscularis propria)	6 (18.8)	5 (5.6)	24 (14.8)	139 (22.9)	
T3b (1-4.9mm beyond muscularis propria)	10 (31.3)	26 (28.9)	58 (35.8)	193 (31.8)	
T3c (5-15mm beyond muscularis propria)	3 (9.4)	29 (32.2)	42 (25.9)	147 (24.2)	
T3d (>15mm beyond muscularis propria)	2 (6.3)	4 (4.4)	11 (6.8)	29 (6.4)	
T4a (invasion of peritoneum)	3 (9.4)	2 (2.2)	12 (7.4)	36 (5.9)	
T4b (invasion surrounding organs/structures)	8 (25.0)	24 (26.7)	15 (9.3)	53 (8.7)	
Positive mesorectal fascia (MRF) or T4 on primary MRI (tumor ≤1mm of the MRF)	19 (59.4)	57 (63.3)	82 (50.6)	281 (46.3)	**0.014**
Mesorectal clinical N-stage					**<0.001**
N0	4 (12.5)	9 (10.0)	30 (18.5)	140 (23.1)	
N1	12 (37.5)	31 (34.4)	75 (46.3)	282 (46.5)	
N2	16 (50.0)	50 (55.6)	57 (35.2)	185 (30.5)	
mrEMVI on primary MRI	11 (34.4)	37 (41.1)	56 (34.6)	210 (34.6)	0.668
Tumor deposits on primary MRI	5 (15.6)	15 (16.7)	33 (20.4)	90 (14.8)	0.483
Neoadjuvant treatment					**0.026**
Short-course radiotherapy	7 (21.9)	26 (28.9)	57 (35.2)	248 (40.9)	
Chemoradiotherapy	25 (78.1)	64 (71.1)	105 (64.8)	359 (59.1)	
Resection of primary tumor					**<0.001**
Non-sphincter sparing (APR/proctocolectomy)	15 (46.9)	54 (60.0)	62 (38.3)	209 (34.4)	
Sphincter sparing (LAR/TME/local excision)	17 (53.1)	36 (40.0)	100 (61.7)	398 (65.6)	
Resection margins (%)					**0.002**
R0	30 (93.8)	74 (82.2)	151 (93.2)	568 (93.6)	
R1	2 (6.2)	16 (17.8)	11 (6.8)	39 (6.4)	
