# Supplementary material for: Prognostic Implications of Lateral Lymph Nodes in Rectal Cancer: A Population-Based Cross-sectional Study With Standardized Radiological Evaluation After Dedicated Training
Source: Dis Colon Rectum. 2023 Jun 1;67(1):42–53. doi: 10.1097/DCR.0000000000002752 (PMC10715698; doi:10.1097/DCR.0000000000002752)
Supplement: Supplementary file 6 [file dcr-67-42-s007.pdf]

Appendix 6. Multivariable analysis of **overall survival** in 891 patients with cT3-4 rectal cancer ≤8cm from the anorectal junction, who were treated with neoadjuvant radiotherapy (short course or chemoradiotherapy). Lateral lymph nodes were included in the analysis based on visibility on primary staging MRI, and stratified for short-axis diameter with a cut-off value of 7mm.

		Univariable analysis			*Multivariable analysis*		
Variable	No.	HR	95% CI	P	HR	95% CI	P
Enlarged (≥7mm) LLN				**0.074**			0.623
No LLN	607	1			1		
≥7mm	122	1.506	1.053-2.153		1.183	0.80671.734	
<7mm	162	1.021	0.709-1.470		0.955	0.660-1.381	
Gender				0.696			
Male	581	1					
Female	310	1.058	0.797-1.404				
Age in years				**<0.001**			**<0.001**
<55	50	1			1		
55-75	472	0.853	0.427-1.702		0.964	0.480-1.936	
75+	369	2.109	1.072-4.151		2.474	1.241-4.934	
Neoadjuvant radiotherapy				0.296			
5x5	338	1					
CRT	553	0.863	0.654-1.138				
Clinical T stage				**<0.001**			**0.002**
T3a	174	1			1		
T3b	287	1.264	0.782-2.043		1.340	0.823-2.180	
T3c	221	1.861	1.158-2.990		1.709	1.044-2.798	
T3d	56	3.201	1.804-5.680		2.501	1.357-4.608	
T4a	53	2.920	1.608-5.304		2.327	1.239-4.369	
T4b	100	3.443	2.076-5.710		2.829	1.617-4.949	
Mesorectal clinical N stage				0.254			
N0	183	1					
N1	400	0.743	0.523-1.057				
N2	308	0.841	0.586-1.207				
Extramural venous invasion (mrEMVI)				**0.033**			0.552
Absent	577	1			1		
Present	314	1.351	1.025-1.782		1.098	0.806-1.496	
Tumor deposits				**0.001**			0.062
Absent	748	1			1		
Present	143	1.735	1.257-2.396		1394	0.983-1.977	
Surgery*				**0.001**			0.060
Sphincter non-sparing	340	1			1		
Sphincter sparing	551	0.641	0.488-0.841		0.754	0.562-1.012	
Margin status				**<0.001**			**<0.001**
R0	823	1			1		
R1	68	3.614	2.519-5.185		2.285	1.556-3.356	
