# Supplementary material for: Prognostic Implications of Lateral Lymph Nodes in Rectal Cancer: A Population-Based Cross-sectional Study With Standardized Radiological Evaluation After Dedicated Training
Source: Dis Colon Rectum. 2023 Jun 1;67(1):42–53. doi: 10.1097/DCR.0000000000002752 (PMC10715698; doi:10.1097/DCR.0000000000002752)
Supplement: Supplementary file 7 [file dcr-67-42-s008.pdf]

Appendix 5. Multivariable analysis of **distant metastases** in 891 patients with cT3-4 rectal cancer ≤8 cm from the anorectal junction, who were treated with neoadjuvant radiotherapy (short course or chemoradiotherapy). Lateral lymph nodes were included in the analysis based on visibility on primary staging MRI, and stratified for short-axis diameter with a cut-off value of 7mm.

		Univariable analysis			*Multivariable analysis*		
Variable	No.	HR	95% CI	P	HR	95% CI	P
Enlarged (≥7mm) LLN				**0.021**			0.389
No LLN	607	1			1		
≥7mm	122	1.590	1.131-2.235		1.268	0.880-1.829	
<7mm	162	1.261	0.907-1.752		1.145	0.821-1.596	
Gender				0.281			
Male	581	1					
Female	310	1.156	0.888-1.506				
Age in years				**0.033**			**0.015**
<55	50	1			1		
55-75	472	0.805	0.462-1.403		1.003	0.570-1.766	
75+	369	1.146	0.656-2.001		1.483	0.835-2.632	
Neoadjuvant radiotherapy				0.731			
5x5	338	1					
CRT	553	1.048	0.803-1.367				
Clinical T stage				**<0.001**			**0.015**
T3a	174	1			1		
T3b	287	1.027	0.666-1.584		0.949	0.611-1.472	
T3c	221	1.785	1.175-2.712		1.456	0.940-2.254	
T3d	56	1.991	1.121-3.537		1.139	0.620-2.094	
T4a	53	3.145	1.858-5.323		2.151	1.231-3.758	
T4b	100	2.790	1.754-4.438		1.567	0.934-2.632	
Mesorectal clinical N stage				0.120			
N0	183	1					
N1	400	1.010	0.705-1.447				
N2	308	1.325	0.924-1.901				
Extramural venous invasion (mrEMVI)				**0.001**			0.166
Absent	577	1			1		
Present	314	1.548	1.195-2.004		1.227	0.918-1.641	
Tumor deposits				**<0.001**			**<0.001**
Absent	748	1			1		
Present	143	2.323	1.737-3.107		1.935	1.406-2.663	
Surgery*				**<0.001**			**<0.001**
Sphincter non-sparing	340	1			1		
Sphincter sparing	551	0.545	0.422-0.705		0.578	0.439-0.762	
Margin status				**<0.001**			**<0.001**
R0	823	1			1		
R1	68	3.235	2.264-4.622		2.265	1.549-3.311	
